# Dual‐Functionality, Bio‐Friendly Artificial Muscles for Actuation and Motion Sensing in Smart Wearable Systems

**DOI:** 10.1002/smll.75219

**Published:** 2026-08-14

**Authors:** Gabriela Ananieva, Cédric Vancaeyzeele, Giao T. M. Nguyen, Frédéric Vidal, Thuan Nguyen Pham Truong, Nils‐Krister Persson, Li Guo, Tommie Varekamp, Felix Dransfeld, Jasper Sikken, Bram Bicknese, Cédric Plesse

**Affiliations:** ^1^ LPPI CY Cergy Paris Université Cergy‐Pontoise France; ^2^ Polymeric E‐textiles University of Borås Borås Sweden; ^3^ Elitac Wearables B.V. Utrecht Netherlands

**Keywords:** auxetic, carbon nanotube (CNT) yarns, electrochemical actuators, e‐textiles, eutectogels, ionotronics, strain sensors

## Abstract

Herein, we report a biofriendly, air‐operating electrochemical platform based on a unipolar coiled carbon nanotube (CNT) yarn artificial muscle that can operate either as an ionic actuator or as an ionotronic strain sensor. The device comprises two coiled CNT yarn electrodes coated with complementary ion‐selective eutectogels, consisting of fixed‐charge polymer networks swollen with a diluted deep eutectic solvent (DES), enabling selective ion transport. Under an applied potential difference, the complementary electrochemical responses of both electrodes produce synchronized unipolar contraction, whereas in sensing mode the device generates a strain‐dependent open‐circuit voltage (OCV) variation. In actuation mode, the device achieves a maximal reversible contractile stroke of 2.9% under a 90 mN pre‐load. In sensing mode, it operates as a self‐powered strain sensor with a sensitivity of 0.5 mV.%^−1^. Poisson–Nernst–Planck modelling is consistent with the proposed mechanism of strain‐induced ion redistribution within the quasi‐solid‐state matrix. Two yarn artificial muscles were woven into a custom‐made, bi‐stretch auxetic structure with a folded zigzag geometry, enabling parallel actuation and strain monitoring, delivering peak‐to‐peak OCV signals of 4 mV at 10% extension. Finally, we demonstrate a wireless wearable armband for real‐time motion monitoring, highlighting the potential of this platform for smart fabrics, wearable electronics, and adaptive soft robotics.

## Graphical abstract

This work presents a biofriendly carbon nanotube yarn artificial muscle operating either as an in‐air unipolar actuator or as a self‐powered ionotronic strain sensor. Using a non‐volatile eutectogel‐derived electrolyte, the system achieves up to 2.9% contraction and 0.5 mV/% strain sensing sensitivity. Integration into auxetic textiles and a wireless armband demonstrates scalable, real‐time physiological motion tracking under ambient conditions.

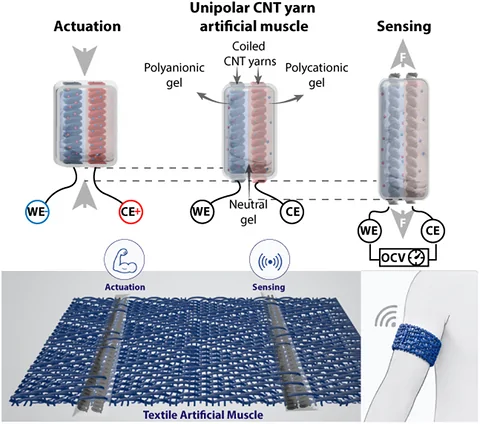

## 1 Introduction

The rapid shift from portable to wearable devices in the fourth industrial revolution has driven growing demand for lightweight, miniaturized, biomimetic technologies, as alternatives to bulky electric motors or pneumatic systems. 1D fiber‐based conductors have emerged as essential building blocks for such systems, given their flexible geometry, scalability, and ease of integration into textile devices [1, 2, 3]. Textile processing could enhance overall actuation performance by additive assembly of actuating yarn as well as mimicking mammal muscles with respect to mechanical compliance [4]. Moreover, overtwisting and coiling specific fibers or yarns enables the creation of high‐performance contractile artificial muscles that, when integrated with sensing or energy‐storage capabilities, offer unparalleled advantages for next‐generation actuating textiles, in comparison to other muscle types, e.g., shape memory materials, electronic electroactive polymers, pneumatic artificial muscles, etc [5]. Depending on the applied physical stimulus, coiled fiber actuators can be classified as thermally‐driven, photo‐activated, vapor absorption‐powered, or electrochemical systems. Thermal actuators based on twisted fishing lines and threads represent one of the earliest breakthroughs, achieving large contractile strokes (> 20%) through volumetric radial expansion during heating [6, 7]. Nevertheless, their reliance on high temperatures, their limited Carnot efficiency, and their slow cooling restrict their practical use, while efforts to add sensing capabilities through core‐shell structures, coatings, or electrospinning are hindered by low conductivity, high power consumption, and durability issues [2, 6, 8]. Similar drawbacks apply to vapor‐ and photoresponsive actuators, which suffer, respectively, from control difficulties and low power densities [7, 9].

Coiled CNT yarn‐based electrochemical actuators have attracted significant attention for overcoming the aforementioned challenges. They operate as fiber‐based supercapacitors, in which charging and discharging of their electrochemical double layers (EDLs) drive relatively fast, low‐voltage actuation (< 5 V). The system architecture typically comprises coiled electroactive yarns and electrolytes, which function as the electrodes and ion sources, respectively. During charge injection, ion‐induced swelling of the yarn electrodes is converted into reversible longitudinal contraction through the coil geometry, enabling precise strain control, high energy density, and good cyclability [8, 9]. CNT yarn electrodes are favored for this purpose due to their high conductivity, high surface capacitance, good mechanical robustness, and lightweight nature [7, 10, 11]. When open‐air actuation is considered, the ion source is typically supplied by a quasi‐solid‐state electrolyte coated onto the surface of the yarns. These electrolytes are typically polymer gels, comprising an organic, aqueous, or ionic liquid electrolyte. Each gel electrolyte category comes with its own limitations, revolving around toxicity for the organic and ionic liquid‐based electrolytes and narrow electrochemical window and poor air stability for the aqueous ones [12, 13, 14]. Recently, deep eutectic solvents (DESs) appeared as a low‐cost, biofriendly alternative to other electrolytes with enhanced stability [13, 15]. In our previous study, we demonstrated for the first time their ability to drive actuation in coiled CNT yarn systems [16]. A biocompatible DES, composed of choline chloride and glycerol, was diluted with water and embedded within non‐toxic polycationic/polyanionic polymer networks, enabling simultaneous contraction of both yarn electrodes in an open‐air (2‐electrode) configuration. In these actuators, the polymer coatings function as ion‐selective membranes, allowing only one type of ion to participate in the intercalation process for each yarn during charging across the entire electrochemical window, resulting in unidirectional and cooperative contraction. These actuators are thus defined by their unipolar stroke. They also showed an enhanced stroke‐to‐charge ratio at higher frequencies compared to bipolar actuators, indicating initial stages of the scan‐rate enhanced stroke (SRES) effect, previously described by Chu et al. [8]. The peak contractile stroke at 90 mN applied load was 3.4% within 30 s when inlaid into a warp‐knitted textile [16].

Beyond actuation, coiled CNT yarn/electrolyte systems can function as passive strain sensors that do not require an external power source. This sensing relies on an ionotronic mechanism, where mechanical deformation is directly transduced into an electrical signal via the movement of mobile ions within the electrolyte [17]. Specifically, the mechanism is based on capacitance modulation: stretching a coiled CNT yarn in an electrolyte alters the ion distribution at the EDL, leading to measurable variations in the open‐circuit voltage (OCV) that directly correlate with strain [18, 19]. For example, a coiled CNT yarn sensor in 0.6 M NaCl aqueous liquid electrolyte generated a peak‐to‐peak OCV of 9.3 mV at 40% applied strain, with a counter electrode designed to remain unstretched to avoid voltage cancellation [19]. To date, such capacitive sensing has been demonstrated only in liquid electrolytes, and its implementation in open‐air 2‐electrode configurations remains largely unexplored. Moreover, the integration of sensing yarns in textiles to form textile‐based artificial muscles for practical applications is done primarily in knits due to their intrinsic compliance and efficient force distribution of the actuator functionality [3, 4]. However, electrochemical coiled yarn actuators, in particular, are challenging to integrate via knitting because they must withstand high stresses and bending forces in the process. Thus, integration techniques were focused on inlaying, where the yarn is incorporated within the loops of a knit as a straight‐line component [16, 20].

Woven techniques offer extensive design freedom through the strategic combination of diverse yarn types. By combining elastic, non‐elastic, and functional yarns in both the warp (longitudinal) and weft (transverse) directions, woven structures can be engineered to exhibit highly tunable deformation behaviours [21]. A key descriptor of this response is Poisson's ratio, PR (ν)—the negative ratio of transverse to longitudinal strain. While conventional woven fabrics typically display a positive PR and contract laterally when stretched, recent advances have shown that certain weave architectures can instead present a negative Poisson's ratio (NPR), or auxetic behavior, in which stretching induces simultaneous lateral expansion [21, 22, 23, 24]. Auxetic textiles offer several advantages: they exhibit synclastic bending for superior conformability to curved surfaces, distribute forces more evenly to reduce local pressure points, and increase air permeability as pores open during extension. These features make them especially promising for performance wear, adaptive structures, and soft robotic systems [24, 25]. Auxetic behaviour has also been shown to improve sensor performance—for example, film‐based strain sensors demonstrating dramatically enhanced stretchability and up to a 24‐fold increase in sensitivity (GF ∼835 at 15% strain) [26]. For garment‐integrated actuators used in haptic feedback or force assistance, auxetic weaves can be particularly advantageous because their high conformability helps maintain consistent actuator–skin contact across complex body geometries. Despite this potential, auxetic woven structures incorporating functional yarns—such as actuators—have not yet been demonstrated.

Building on our recently developed biocompatible unipolar coiled CNT/eutectogel artificial muscles, this study expands the functionality of the underlying electrochemical platform beyond actuation. We demonstrate that the same unipolar coiled CNT yarn can be configured to provide either tunable open‐air actuation or ionotronic strain sensing. Crucially, we further show that unipolarity is the key design principle enabling stable open‐air sensing through strain‐induced electrochemical potential changes in a solid‐state electrolyte, establishing a sensing mechanism that has previously remained inaccessible in other reported air‐operating CNT yarn systems. To demonstrate the versatility of this platform, the unipolar CNT yarn devices were integrated into a custom bi‐stretch auxetic weave. This fabric architecture leverages differential shrinkage to achieve both wearer comfort and heightened mechanical sensitivity. Finally, we demonstrate a battery‐powered wireless wearable armband that combines electrically driven textile actuation with embedded strain sensing for real‐time monitoring of physiological motion. Together, these results establish a modular textile‐compatible platform in which unipolar CNT yarn artificial muscles and open‐air ionotronic strain sensors share a common electrochemical architecture. The integration of this platform into auxetic textiles provides a pathway toward next‐generation electroactive textiles, wearable electronics, and adaptive soft robotic systems.

## 2 Results and Discussion

The unipolar solid‐state artificial muscles were fabricated by assembling two coiled CNT yarns in a parallel, two‐electrode configuration. Each yarn was coated with a charged polyelectrolyte layer designed to selectively block the movement (intercalation) of one type of ion during electrical stimulation. Because the two yarn electrodes experience opposite polarization during actuation, their coatings must carry opposite charges to enable both yarns to contract simultaneously. Specifically, a yarn coated with a negatively charged (polyanionic) polymer, which allows cation movement, promotes cathodic contraction, while a yarn coated with a positively charged (polycationic) polymer, allowing anion movement, contracts anodically [8, 16, 27]. The two coated yarns must then be connected by a suitable gel electrolyte to form an electrochemical cell equivalent. A neutral gel with both mobile cations and anions was used for that purpose.

The gels were synthesized in the presence of a biocompatible deep eutectic solvent (DES)‐based electrolyte as previously reported by Ananieva et al. [16], chosen as a sustainable alternative to conventional organic and ionic liquid electrolytes. Briefly, this DES consists of choline chloride and glycerol, acting, respectively, as the hydrogen bond acceptor (HBA) and the hydrogen bond donor (HBD), in a 1:2 molar ratio, into which water (HBA:H_2_O = 1:16 molar ratio) is added to reduce viscosity.

The electrolyte gel was composed of a polymer network (50 wt.%) in a DES‐based electrolyte. Three polymer networks were considered:

1. A polyanionic network synthesized from 3‐sulfopropyl acrylate potassium salt (SPA), providing negatively charged (─SO_3_^−^) groups.
2. A polycationic network synthesized from 2‐acryloxyethyltrimethylammonium chloride (AETA), providing positively charged (─N(CH_3_)_3_^+^) groups.
3. A neutral network synthesized from 2‐hydroxyethyl acrylate (HEA).

All polymers were crosslinked using polyethylene glycol diacrylate (PEGDA) (4 wt.% of the polymer network), and the syntheses were initiated by a non‐cytotoxic photoinitiator 2‐hydroxy‐4′‐(2‐hydroxyethoxy)‐2‐methylpropiophenone (1 wt.% of the polymer network) [28].

The gel precursors were applied in two coating steps—one before coiling and one after. Following coating, the coiled yarns were UV‐irradiated to complete network formation. Two yarns, one carrying a polyanionic gel and the other a polycationic gel, were then aligned in parallel and encapsulated within a neutral gel to form the final actuator assembly.

We previously demonstrated that symmetric, synchronized actuation of both yarns was achieved through ion transport and insertion within the two polymer‐coated yarn electrodes [1]. From an electrochemical point of view, charge insertion is balanced between the two electrodes, as the actuator operates in a two‐electrode configuration. The identical deformation of the two yarns arises from ion insertion with a similar ion size to compensate for equivalent charge at the two electrodes. Specifically, the hydrodynamic radius of the choline ion is approximately 2.5–3.5 Å, while chloride exhibits an effective solvated hydrodynamic radius of approximately 3.0–3.2 Å, which is substantially larger than its crystallographic radius (1.81 Å [2]) owing to strong hydrogen bonding with glycerol and water [3, 4]. The crystallographic radius of the choline ion is 3.29 Å, in the range of its hydrodynamic radius [5]. Additional information is available in the Supporting Information, where further insights into the nearly identical transport properties of the two ions are presented. These properties account for the highly symmetric and synchronized actuation observed in both yarns, as reported in our previous study.

Figure 1 illustrates the operating principles of the unipolar artificial muscle and its integration into a textile structure.

**FIGURE 1:**
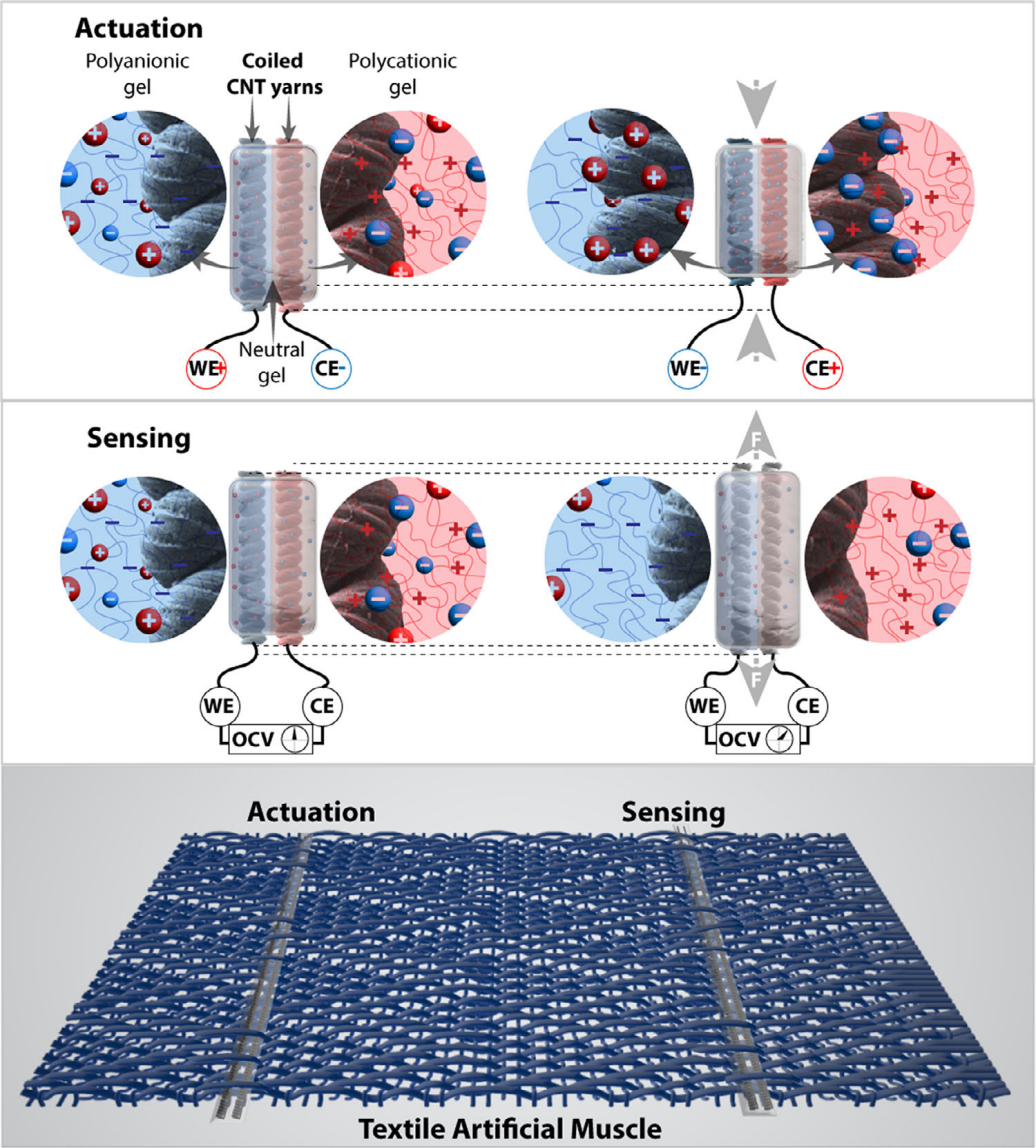
(A) Actuation principle of the unipolar solid‐state artificial muscle—tensile contraction induced by ion influx during charge injection, with the coiled CNT yarns functioning as the working (WE) and counter (CE) electrodes; (B) Sensing principle of the unipolar quasi‐solid‐state artificial muscle—applied tensile strain causes ion efflux and densification of the yarn, resulting in measurable voltage variation.

### 2.1 Bimodal Operation of the Unipolar Artificial Muscles in Open Air

Actuation was characterized using a potentiostat and a lever arm system. Charge was injected into the yarns serving respectively as WE and CE, and the resulting contraction was measured under an applied constant force of 90 mN. For samples longer than 50 mm, actuation strain was quantified from high‐resolution video recordings by tracking predefined reference points using motion‐tracking software and calculating their relative displacement. In this setup, the applied load was suspended from the bottom end of the actuator. The electrochemical stability of the ChCl/glycerol eutectogel‐coated CNT yarn system was evaluated by cyclic voltammetry over potential windows ranging from −3.8 to +3.8 V at 50 mV s^−1^ and −5 to +5 V at 1000 mV s^−1^ (Figure S1). The artificial muscle is equivalent to a two‐carbon‐electrode electrochemical cell. The wide electrochemical stability window previously reported for carbon electrodes is generally attributed to kinetic rather than thermodynamic effects [29]. Although the thermodynamic decomposition potential of water is 1.23 V, the hydrogen and oxygen evolution reactions on carbon electrodes are kinetically hindered because of their high overpotentials. As a result, these systems can operate at significantly higher cell voltages without substantial water electrolysis. A similar explanation has been proposed for high‐voltage aqueous carbon‐based supercapacitors, in which the practical electrochemical stability window extends well beyond the thermodynamic limit [30].

Chronoamperometry (CA) with potential steps of 3 and −3 V, and a period of 60 s was performed to assess the device's performance under conditions representative of practical use (Figure 2A).

**FIGURE 2:**
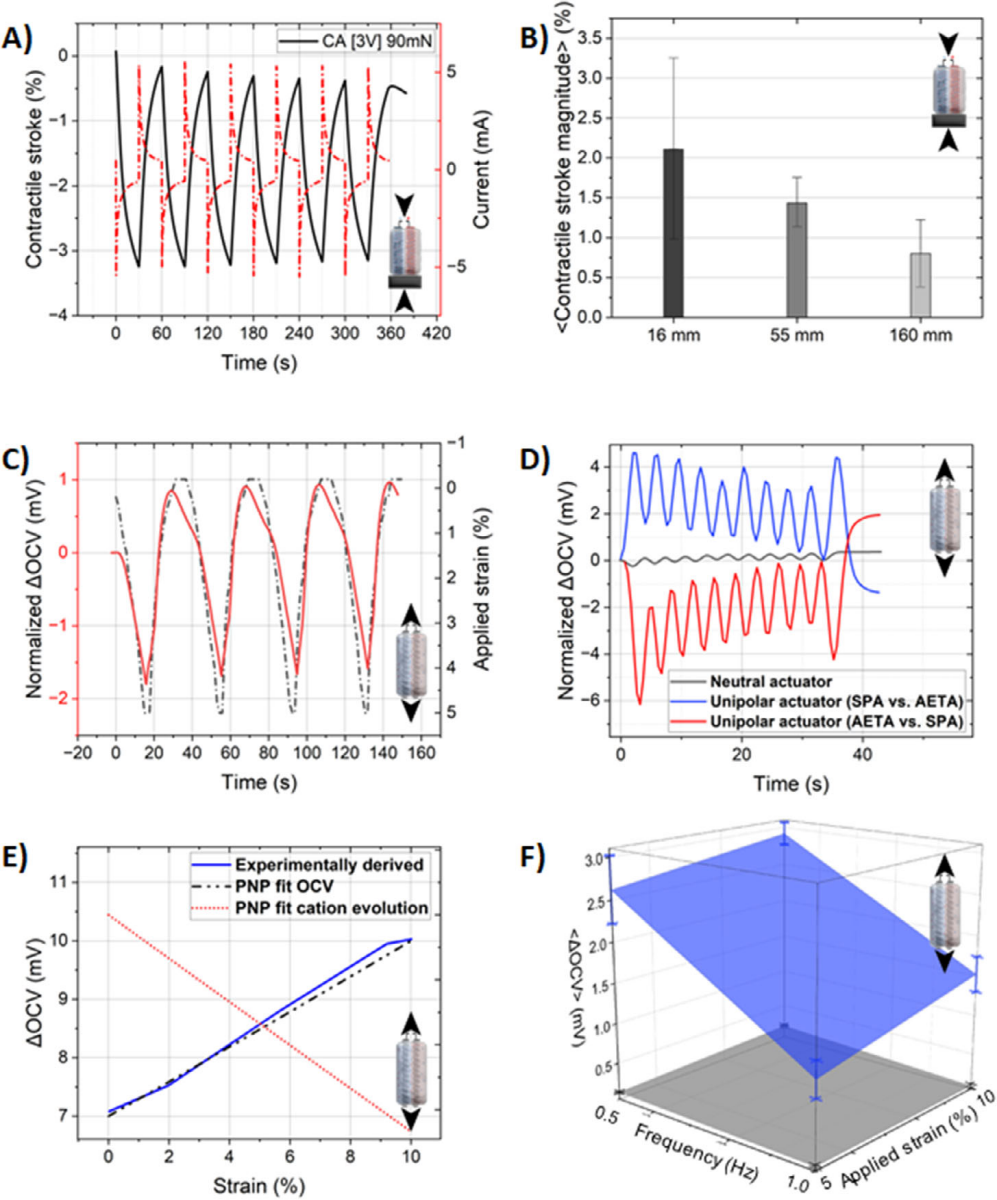
(A) Contractile stroke of the unipolar solid‐state actuator (16 mm) in open air during CA with potential steps of ± 3 V, 60 s period, and 90 mN applied tensile force; (B) Dependence of contractile stroke on actuator length, measured under the same CA conditions. Values represent the average of at least three independently fabricated actuators per length, with error bars corresponding to the standard deviation; (C) Sensing response (OCV variation) of the unipolar solid‐state actuator subjected to 5% tensile strain at a rate of 10 mm min^−1^, with 3 s retention in relaxed state; (D) Comparison of OCV variation at 10% tensile strain (0.5 Hz) between a neutral gel‐coated control artificial muscle and the unipolar charged‐gel one. Measurements were conducted with the polyanionic (SPA) yarn as WE vs. the polycationic (AETA) yarn as CE (noted unipolar actuator (SPA vs. AETA)) (blue), and vice versa (red); (E) OCV variation of the unipolar actuator (SPA vs. AETA) under 0%–10% applied strain, showing the fitted PNP curve and the corresponding hypothetical cation concentration; (F) OCV variation of the control and unipolar actuators as a function of strain magnitude and strain rate. Data points represent the mean of 5 consecutive cycles and error bars indicate the 95% confidence interval (CI).

The time‐dependent actuation response is presented in Figure 2A, showing a mean contractile strain of 2.9% across all cycles. The actuation is fully unipolar, as expected from the presence of the charged polyelectrolyte coatings [16, 27]. The operating current remains low (∼5 mA at peak) and decreases rapidly over time, which is advantageous as it minimizes Joule heating and facilitates seamless integration into wearable systems, where such currents can be directly sourced from a microprocessor. The average contractile stroke of coiled yarns as a function of their length, measured from at least three independently fabricated actuators per length, along with the corresponding standard deviation, is summarized in Figure 2B. For samples shorter than 20 mm, the mean contractile stroke was the highest, reaching 2.1%.

To evaluate the structural underlying causes of performance scaling, optical profiling of the fully hydrated gel coating was performed along the yarn lengths (Figure S2). The mean coating thickness bridging the electrode elements was determined to be 1.9 mm. For shorter samples (< 20 mm), the coating exhibited higher structural homogeneity, maintaining a tight standard deviation of 0.01. However, scaling the sample length complicated manual processing, causing the coating thickness standard deviation to rise to 0.04 for samples longer than 150 mm.

This spatial non‐uniformity can introduce localized fluctuations in electrochemical impedance and ion diffusion dynamics along the yarn surface, thus reducing local actuation efficiency. When compounded by minor variations in linear density and Ohmic losses over extended conductive pathways, these factors systematically diminish the net macro‐scale stroke of uninterrupted longer specimens. Nevertheless, the contraction remains visibly perceptible even at longer actuator lengths, as demonstrated in Video S1, where the yarns have a length of 55 mm.

This structural correlation highlights the implementation of automated, continuous‐feed coating systems as a critical trajectory to ensure flawless homogeneity during large‐scale fabrication.

Importantly, this length‐dependent performance scaling does not present a fundamental barrier to macro‐scale textile viability, but rather defines the architectural rules for smart textile design. Woven or knitted textile geometries inherently lend themselves to parallel network topologies and modular structural design. For instance, integrated networks of shorter, high‐performance active segments can be arranged in parallel or series configurations, thereby preserving the peak contractile stroke of optimized 20 mm elements while scaling the total output to macroscopic dimensions. Furthermore, the textile matrix permits the interleaving of highly conductive, auxiliary yarn pathways to serve as localized electrical feeders (bus bars). By distributing the voltage input at strategic periodic intervals along an extended yarn, the linear Ohmic gradient can be effectively neutralized, ensuring uniform electrochemical activation across large areas.

Next, the sensing capabilities of the artificial muscles were evaluated by measuring the OCV while applying controlled strain using a tensile machine. The yarns were connected as the working (WE) and counter (CE) electrodes of the potentiostat, analogous to the configuration used during actuation measurements (Figure 1B).

When the polycationic gel (AETA)‐coated yarn served as the working electrode (WE), the observed peak‐to‐peak OCV was 2.5 mV under 5% tensile strain, applied at 10 mm/min with a 3 s hold at the relaxed state to allow some charge re‐equilibration (Figure 2C). This corresponds to a sensitivity of 0.5 mV %^−1^, making it more responsive than comparable systems operated in liquid electrolytes (∼0.23 mV %^−1^), where voltage variation arises from stretch‐induced changes in the electrochemical double‐layer capacitance (EDLC) caused by ion movement [19].

To better understand the underlying sensing mechanism, the electromechanical response of the unipolar artificial muscle was compared to that of a control actuator fabricated in the same way but using only the neutral gel for electrodes and to glue the electrodes. The results are presented in Figure 2D, where both muscles were stretched to 10% at a frequency of 0.5 Hz. The neutral‐gel actuator exhibits only negligible OCV variation, as expected for a two‐electrode symmetric configuration in which identical ion redistribution occurs at both yarns during mechanical stimulation. As a result, the potential of each yarn varies similarly, effectively cancelling any OCV difference. The small residual signal observed is therefore likely attributable to minor asymmetries between the two yarn electrodes. In contrast, the unipolar artificial muscle exhibits approximately a 15‐fold enhancement in peak‐to‐peak OCV, independent of whether the polycationic (AETA) or polyanionic (SPA) yarn functions as the WE. Notably, interchanging the roles of the WE and CE between the oppositely charged yarns produces a mirrored OCV response. When the polycationic yarn serves as the WE, the OCV becomes increasingly negative with applied strain; conversely, when the polyanionic yarn acts as the WE, the OCV increases positively and proportionally with strain (Figure 2E). This behaviour, drastically different to the one obtained with neutral gel, can be explained by the presence of fixed charges on the polymer gel backbone through the electrochemical double‐layer theory, first proposed by Helmholtz‐Perrin and later refined by Gouy‐Chapman and Stern. The electrode–electrolyte interface remains electrically neutral under all conditions, maintaining equal and opposite net charge densities [31]:

$$Q_{electrode}=-Q_{solution}$$

Since the system operates under open‐circuit conditions (OCV), no external current is applied. The grafted (fixed) charges within the polymer gel define the intrinsic charge on the electrode side, while the mobile ions in the electrolyte form the oppositely charged layer in solution. Thus, the interfacial charge balance can be expressed as:

$$Q_{electrode}=-\left(Q_{fixed}+Q_{mobile}\right)$$

When the yarn is stretched, the densification of the coiled yarn expels some of the mobile counter ions. For the polyanionic (SPA‐coated) yarn, these mobile ions are cations, and tensile strain therefore reduces the local cation concentration, meaning:

$$\Delta Q_{mobile}\;<\;0$$

Because *Q_fixed_* remains constant, the corresponding variation in the electrode charge is:

$$\Delta Q_{electrode}=-\Delta \;\left(Q_{fixed}+Q_{mobile}\right)=-\Delta Q_{mobile}$$

Here, since *∆Q_mobile_* < 0, Δ*Q_electrode_* > 0, which means the electrode becomes more positively charged (its potential shifts upward).

This explains why the polyanionic electrode shows an increase in OCV when strain is applied. The effect can be understood by recognizing that mechanical deformation shifts the ionic equilibrium at the electrode interface. A similar strain‐induced response occurs at the polycationic counter‐electrode, but with opposite changes in charge and potential. The measured OCV is therefore the difference between these two opposing contributions, which results in a non‐zero OCV.

To provide a continuum electrostatic interpretation of the experimentally observed strain‐dependent OCV response, a one‐dimensional steady‐state Poisson–Nernst–Planck (PNP) framework was employed. The model was not intended to establish the mechanism independently, but rather to examine whether the experimentally observed voltage trends are consistent with strain‐induced ion redistribution at the electrode–electrolyte interface. This continuum model couples the electrostatic potential (*φ*) to the spatial distribution of mobile ions (*c*_1_, *c*_2_) within the polyanionic gel.

The nondimensional PNP system, following the scaling approach by Chao, Geng, and Krasny [32], is given by the modified Poisson Equation (1) and the steady‐state Nernst–Planck Equation (2):

(1) $$-\varepsilon \;\varphi^{\prime \prime }=\sigma_{2}\;\left(z_{1}c_{1}+z_{2}c_{2}+\rho_{f}\right)$$

(2)

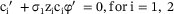

Here φ′′ is the electrostatic potential scaled by the thermal voltage ($\varphi_{0}=\frac{k_{B}T}{e}\;\approx 25.7\;mV$), ε is the dielectric permittivity (treated as spatially uniform), *c*_1_ and *c*_2_ are nondimensional ion concentrations, scaled by the bulk electrolyte concentration. The anions have valences (*z*_1_ = ‐1) and the cations (*z*_2_ = 1). ρ_*f*_ is the nondimensional fixed charge density of the negatively charged polymer. Thus, the term (*z*_1_ *c*_1_ + *z*_2_ *c*_2_ +  ρ_*f*_) represents the total charge density at any position x. Since ρ_*f*_ < 0, local electroneutrality requires a higher cation concentration (*c*_2_) than anion concentration (*c*_1_) inside the gel. The concentration gradient (*c_i_* ′) and the electric field (φ′) are coupled so that ions redistribute to minimize the internal electrostatic energy.

The coefficient σ_1_ ≈ 1 controls the balance between ion diffusion and electromigration, while σ_2_ scales the strength of the coupling between the charge density and the potential curvature. Full definitions of σ_1_ and σ_2_ are provided in the Supporting Information.

Within the model, mechanical strain is assumed to modify the electrostatic environment through two constitutive relations. The effective fixed charge density is assumed to increase proportionally with strain due to densification of the gel within the tightening CNT yarn structure, which increases the concentration of immobile anionic sites per unit volume. Simultaneously, the dielectric permittivity is assumed to decrease slightly with strain [33, 34], owing to partial expulsion of the water–glycerol solvent during densification. Because water and glycerol possess substantially higher dielectric permittivity than the polymer network, this solvent redistribution is expected to reduce the effective dielectric screening. These phenomenological assumptions were introduced to investigate whether strain‐induced changes in the electrostatic environment are sufficient to reproduce the experimentally observed OCV response. The dielectric relationship ε(strain) = ε_0_(1 − 0.2 × strain) was selected as a conservative approximation, providing a modest correction to dielectric screening rather than acting as a dominant fitting parameter. Sensitivity analysis confirmed that variations in this term had only a minor effect on the predicted OCV, while preserving the predicted voltage polarity and increase in OCV with strain.

Under these conditions, the coupled Equations (1) and (2) were solved numerically. The OCV was then calculated as the potential difference between the bulk‐averaged regions of the working and counter electrodes and converted back to physical volts using the thermal voltage: φ_0_ Equation (3):

(3) $$\mathrm{OCV}=\left({\langle \varphi \rangle }_{\mathrm{WE}-\mathrm{side}}-\;{\left\langle \varphi \right\rangle }_{\mathrm{CE}-\mathrm{side}}\right)\varphi_{0}$$

The model output was linearly mapped to the experimental 7–10 mV range, preserving the predicted voltage polarity and strain‐dependent OCV slope (Figure 2E). The complete model derivation can be found in the Supporting Information with all implemented parameters in Table S1. Future studies combining the model with high‐resolution operando ion‐mapping techniques could enable direct real‐time visualization and predictive quantification of strain‐induced ion redistribution within the system.

Finally, the electrochemical response was measured as a function of strain percentage and frequency. The average OCV values (polyanionic electrode relative to the polycationic) are shown in Figure 2F. As expected, the OCV amplitude is lower at higher frequencies (1 Hz) and lower strain percentage (5%), reaching an average value of 1.1 mV (CI ± 0.2). Applying a higher strain of 10% at the same frequency (1 Hz) leads to an OCV increase to 1.5 mV (CI ± 0.2), and reducing the frequency to 0.5 Hz at this strain percentage leads to the highest peak‐to‐peak OCV of 2.9 mV (CI ± 0.15). Stretching the sample 5% at 0.5 Hz creates a 2.6 mV (CI ± 0.4) OCV variation. These results show consistent sensing behaviour, able to differentiate between different strain magnitudes and rates.

Consequently, the bio‐friendly artificial muscles manufactured using coiled CNT yarn demonstrate properties for actuation and motion sensing.

To enable integration into smart wearable devices, the dual‐functional unipolar artificial muscle was incorporated into a purpose‐designed woven textile. The woven architecture described in the following section was engineered specifically to complement the actuator's deformation mode: its auxetic, folded‐zigzag geometry accommodates the muscle's longitudinal contraction and radial expansion, while providing mechanical stability, strain redistribution, and dedicated channels for embedding the yarn‐based device. In this way, the textile platform transforms the artificial muscle from a standalone component into a functional element of a larger, adaptive material system.

### 2.2 Design and Characterization of a Woven Textile for Artificial Muscle Integration

Woven fabrics are formed by interlacing two orthogonal yarn systems: warp (longitudinal) and weft (transverse). Compared to knitted structures, whose interlooped yarns provide high elasticity and conformability, woven fabrics offer greater dimensional stability and shape retention due to the restricted mobility at yarn crossover points. Their key advantage, however, is customizability. Computer‐assisted handlooms allow precise, low‐tension insertion of diverse yarn types and deliberate variation in thread density and interlacement patterns. This design freedom enables the development of woven fabrics with unconventional mechanical responses, including a negative NPR, where the fabric expands transversely when stretched longitudinally. Woven NPR/auxetic textiles provide a uniquely adaptable platform that combines ease of functional‐yarn integration with high conformability, making them particularly suited for applications requiring controlled deformation and body‐adaptive behaviour [21, 22, 23, 24]. Accordingly, the goal was to develop an auxetic woven structure, which would complement the artificial muscle's performance.

NPR wovens can be realized either by incorporating auxetic yarns into conventional weave geometries or by designing auxetic weave architectures using conventional yarns. Zulifqar et *al*. (2018) demonstrated that combining conventional yarns with contrasting elastic moduli within a geometry predisposed to auxetic behavior, such as a foldable zigzag, produces NPR (Figure 3A) [24]. In this structure, the unit cell acts as a mechanically coupled system: longitudinal stretching forces the folded regions to open, producing net transversal expansion [22, 23, 35]. This architecture is typically created by alternating regions of high and low interlacing density. Tight segments are formed using a plain weave (a 1/1 interlacing pattern), while loose segments utilize floats, such as a 4/1 satin or a 3/1 twill weave. Elastic yarns function as return springs that restore the structure, while non‐elastic, i.e., rigid, yarns provide stability. Under tension, the warp and weft yarns first straighten and flatten the folds before aligning, generating NPR behavior, commonly reaching around −0.1 at moderate strains. The NPR magnitude depends strongly on factors such as the ratio of elastic to rigid yarns, float length, and the direction of applied strain [22, 23, 24, 35].

**FIGURE 3:**
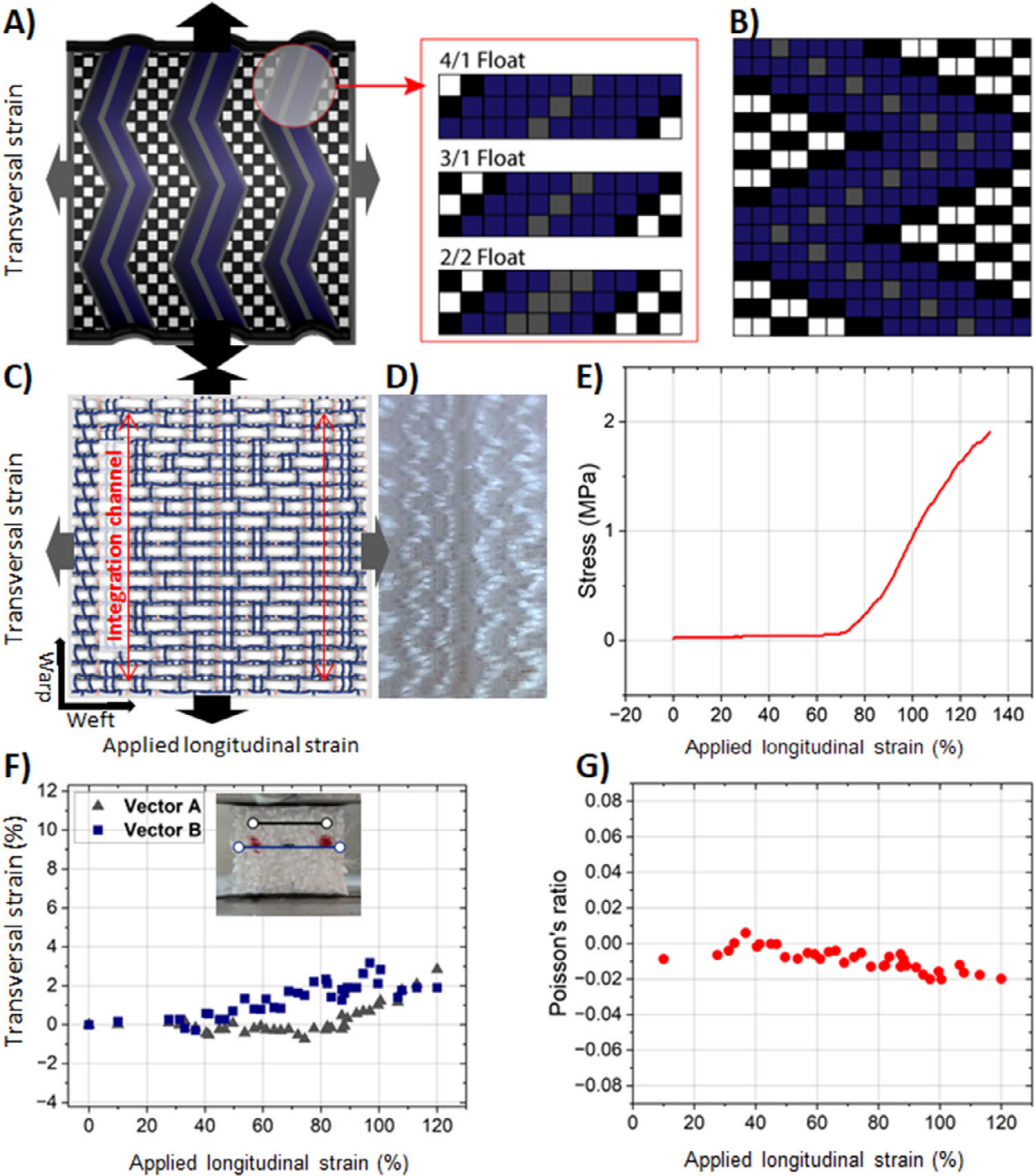
(A) A schematic of the working principle of woven auxetic textiles, consisting of a flexible float component and a rigid plain weave one; (B) Peg plan of the folded‐geometry unit cell developed in this study (4/1 float and plain backed, 2/2 twill), where dark squares indicate warp yarns passing over weft yarns; (C) A 3D rendering of the textile architecture, showing elastic yarns in dark blue, inelastic warp yarns in pale pink, and inelastic weft yarns in white. Longitudinal stretch is applied along the warp direction, corresponding to the actuator integration channels highlighted in red; (D) Photograph of the completed woven structure; (E) Stress–strain behavior of the developed weave and corresponding Young's modulus; (F) Evolution of the fabric width with applied longitudinal strain, analyzed via tracking of two lateral vectors (A and B) as illustrated in the inset graph; (G) Average Poisson's ratio as a function of applied longitudinal strain, derived from the mean of vectors A and B.

In this study, a bi‐stretch woven fabric was engineered as a functional substrate for integrating artificial muscles, combining high conformability with excellent dimensional stability. The chosen design employed a folded zigzag auxetic geometry, optimized to operate from a fully stretched (decrimped) state—matching the actuation mode of the artificial muscle since the muscle contracts under electrical stimulation. This structure enables efficient mechanical coupling between the fabric and actuator, as the crimping and decrimping of the woven floats mimic the actuator's radial expansion and longitudinal contraction during operation. The elastic yarns incorporated into the structure enhance local deformation accommodation and ensure full recovery after extension.

The fabric was woven on a computer‐assisted 24‐shaft dobby handloom using a straight draft. The auxetic geometry consisted of a 4/1 float (soft component) and a hybrid plain‐backed twill, composed of 1/1 interlacements in the warp and 2/2 floats in the weft (rigid component) (Figure 3B). This hybrid pattern created linear channels along the zigzag fold, intended to accommodate the yarn‐based actuators (Figure 3C). The warp consists of a 3:1 ratio of elastic to non‐elastic yarns, arranged at a density of 10 threads per centimeter. The hand‐inserted weft follows a 1:1 elastic to non‐elastic pattern. In the 3D model in Figure 3C, elastic yarns are depicted in dark blue, the inelastic warp in pale pink, and the inelastic weft in white. A photograph of the fabricated fabric is shown in Figure 3D. The elastic yarns exhibited an elongation at break exceeding 250% and a Young's modulus of 3.3 MPa, while the non‐elastic warp and weft yarns displayed moduli of 349 and 294 MPa, respectively—values consistent with synthetic multifilament yarns characterized by moderate internal porosity and crimp. The high biaxial shrinkage induced by the elastic components promoted crimp formation in the non‐elastic yarns, producing surface floats that contributed to the fabric's textured morphology. Under longitudinal stretching along the warp direction—which corresponds to the zigzag axis—the fabric exhibited a pronounced toe region up to approximately 70% strain, associated with progressive uncrimping of the floats. Beyond this region, the mechanical response transitioned into a linear regime dominated by yarn alignment and elastic extension (Figure 3E).

To evaluate the lateral deformation behavior, two vectors, A and B, were defined along the weft direction, as shown in the inset of Figure 3F, while the longitudinal strain was applied along the warp. Because woven fabrics typically exhibit seating and drift during the first loading cycle, the third loading cycle was analyzed as the stabilized response. Each vector was normalized with respect to its length at the onset of longitudinal loading. Vector A, positioned closer to the top clamp and not spanning the full fabric width, exhibited a mixed response. Its magnitude remained nearly constant at lower loads and began to expand only at higher strains. This delayed expansion arises from the fabric's high overall elasticity and the structural asymmetry between warp and weft yarns. The weft floats, being thicker and more crimped than the warp yarns, straighten (“unfold”) more slowly, so the early stages of deformation are dominated by warp alignment, while the weft contributes more significantly at higher loads. In contrast, vector B, which spans horizontally across the entire fabric width, experienced stronger boundary constraints. The clamping pressure held the fabric taut locally, restricting complex local yarn rotations and enforcing a more uniform, global deformation. As a result, the deformation pattern near the grips showed a predominantly linearly increasing lateral strain, as the overall fabric structure opened under tension. Minor surface irregularities and local slip events introduced small fluctuations (“noise”) in this dataset—a common observation in tensile tests of woven materials due to grip‐induced micro‐slippage. Overall, the edge constraint simplified the deformation field compared to the more heterogeneous internal regions.

The effective Poisson's ratio ν_*LT*_ was calculated according to Equation (4), where ε_*T*_ is the transverse strain along the weft and ε_*L*_ represents the longitudinal strain along the warp, calculated from the averaged strain data along vectors A and B.

(4) $$\nu_{LT}=-\frac{\varepsilon_{T}}{\varepsilon_{L}}$$

The Poisson's ratio, reported as a function of applied longitudinal strain, remained approximately zero up to about 70% strain, after which it transitioned to negative values, reaching around −0.02 as the zigzag geometry became fully unfolded (Figure 3G). This indicates that the initial deformation is governed primarily by internal geometric rearrangement and yarn realignment, whereas the high‐strain regime shows the onset of auxetic behavior driven by the straightening of the weft floats. The delayed emergence of the NPR effect compared to previous studies (e.g., Cao et al. [35]) is attributed to the combined influence of higher yarn elasticity, lower thread density, and the hybrid 1/1–2/2 interlacement pattern employed in this work, which collectively moderate the structural unfolding dynamics.

Notably, when the same fabric was tested under relaxed boundary conditions—where the grips were positioned outside the woven frame, allowing full decompression of the elastic weft—a visibly stronger lateral expansion was observed (Figure S3). Although this configuration amplifies the apparent auxetic effect, part of the observed motion originates from elastic recovery of the pre‐shrunk weft yarns rather than purely geometric unfolding. Thus, both mechanisms—elastic decompression and auxetic decrimping—contribute to the overall transverse expansion. These results highlight the strong influence of boundary constraints on the apparent NPR response of highly elastic woven systems. A more detailed statistical and structural analysis of the fabric's deformation behavior under longitudinal strain is detailed in Figure S3, while the measured Poisson's ratio is contextualized in Table S3 through comparison with representative literature values of woven auxetic structures.

Overall, the developed bi‐stretch woven architecture demonstrates a strain‐dependent auxetic behavior arising from the interplay between yarn elasticity, weave geometry, and inter‐yarn friction. The hybrid 1/1–2/2 weave enhances conformability while maintaining mechanical integrity, and the alignment of floats with the zigzag fold creates continuous channels that would facilitate efficient mechanical coupling with the artificial muscles. It is expected that during actuator contraction, the associated radial expansion would induce re‐crimping of the weft floats, resulting in a synergistic biaxial contraction of the textile and enhanced motion sensitivity. The inherent auxetic mechanism is expected to improve conformability to curved surfaces, enhance load distribution, and reduce localized stress accumulation during repeated deformation—key advantages for wearable and adaptive textile systems. Together, these properties establish a durable, dimensionally stable, and responsive substrate suitable for integration with soft actuation and sensing components.

### 2.3 Stand‐Alone Wearable Textile‐Based Artificial Muscles

Two open‐air operating, unipolar, coiled CNT yarn artificial muscles (length = 15 cm) were fabricated and integrated into two parallel channels aligned with the zigzag folds of the auxetic textile, resulting in a textile‐based artificial muscle with an overall width of 1.6 cm and length of 20 cm including selvages. A close‐up view of a section of the integrated structure is shown in Figure 4A. Actuation was driven electrochemically, with the polyanionic‐coated CNT yarns connected to the working electrode (WE) and the polycationic‐coated CNT yarns to the counter electrode (CE).

**FIGURE 4:**
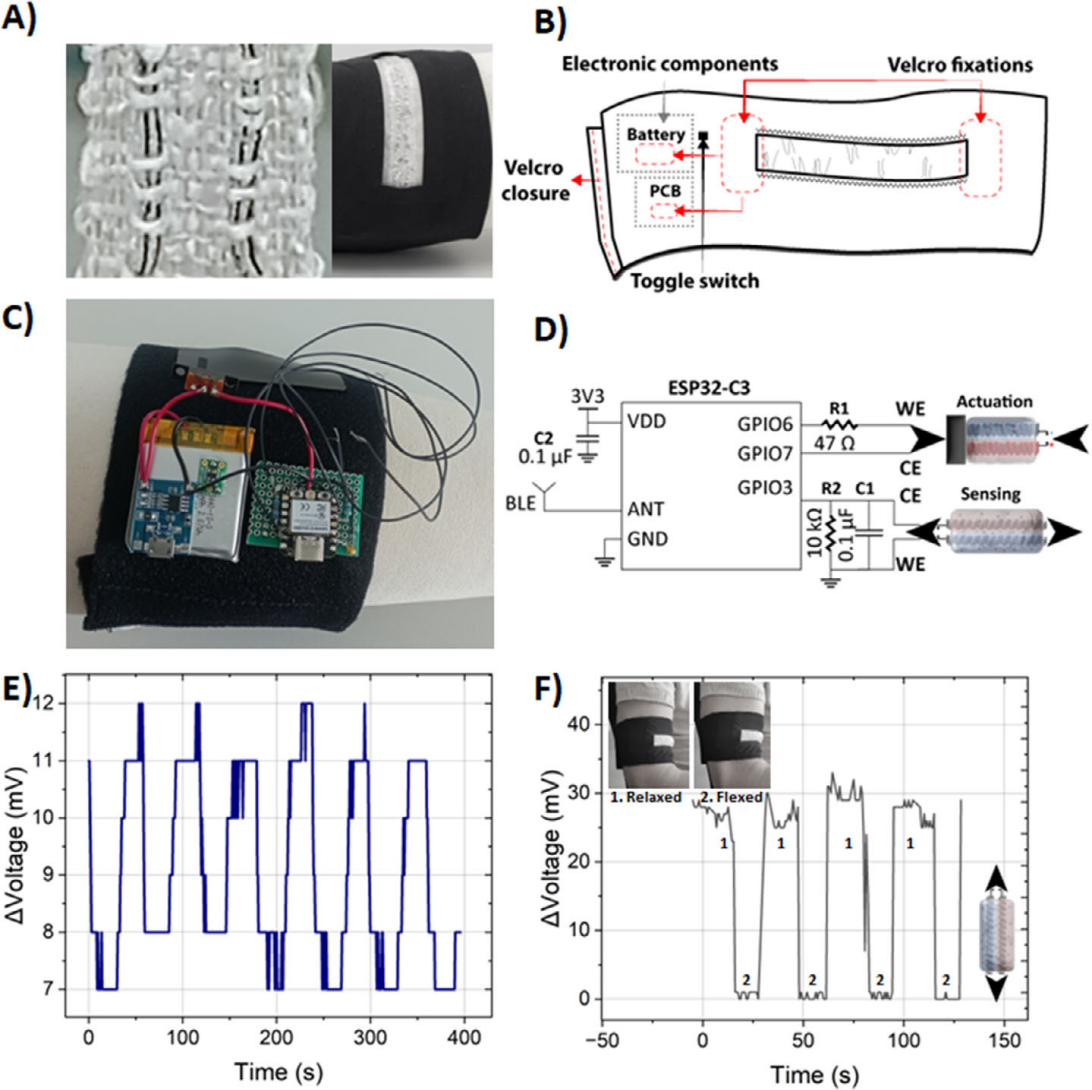
(A) A close up of the integrated artificial muscles in the auxetic textile substrate and the smart wearable; (B) Technical drawing of the armband illustrating the overall design and structural configuration; (C) Electronic circuitry components comprising the ESP32‐C3 microcontroller, power unit, and passive components, attached to the loop fabric (inner layer) using Velcro hooks to enable modular integration and easy detachment; (D) Circuit schematic illustrating the interconnection between the actuator, sensing unit, and electronic components; (E) Sensing results of the device when subjected to stretch/release cycles; (F) Real‐time monitoring results of muscle flexion, measured with the smart wearable device and transmitted wirelessly.

Next, the textile‐based artificial muscle was integrated into a battery‐powered wearable system specifically designed for this purpose. The wearable device took the form of an adjustable armband composed of elastic synthetic textiles, including a nylon tricot jersey knit outer layer and an inner loop fabric compatible with a hook (Velcro) fastening (Figure 4B). The inner loop layer enabled modular placement and repositioning of electronic components, which were attached via hook segments. The textile‐based artificial muscle was secured in a pre‐stretched state within a ripstop‐fabric tunnel using hook attachments on both ends. This tunnel not only facilitated easy replacement of the actuator but also served as a protective barrier against mechanical wear and environmental exposure. The entire armband could be fastened and size‐adjusted using the Velcro closure, ensuring a stable and comfortable fit around the biceps.

The armband contained a 3.7 V Li‐ion battery with a TP4056 charger, a power toggle switch, and a Seeed Studio XIAO ESP32‐C3 microcontroller (Figure 4C). The system was designed to provide both sensing and actuating functionality on the same circuit board. The actuating artificial muscle was driven from GPIO6 and GPIO7 as alternating WE/CE terminals, switching polarity every 30 s to mimic potentiostatic cycling with potential steps of ± 3 V. The sensing muscle was connected to GPIO3 and ground for passive voltage tracking during deformation (OCV‐like measurement). To ensure stable operation, resistors and capacitors were integrated at key nodes to suppress excessive current transients generated during polarity switching. The wiring scheme is shown in Figure 4D. Processed voltages were transmitted wirelessly to a Python acquisition interface for real‐time logging and visualization.

The 15 cm long artificial muscles could achieve a mean contractile stroke of 0.9 ± 0.2% at 90 mN applied force, when tested on their own. In the textile, they promoted reduced but visible displacements even under an applied tensile load as high as 2 N, applied by suspending calibrated weights from one end of the textile specimen. These deformations were not linear due to differences in the actuation performances of the two artificial muscles, thus highlighting the importance of strain and speed uniformity when coupling actuators.

Conversely, when operated as a strain sensor—subjected to 10% cyclic strain relative to a pre‐stretched baseline—the auxetic textile‐integrated muscle demonstrated performance comparable to individual coiled CNT yarns. Notably, its sensitivity was markedly superior to that of an identical muscle embedded in a satin weave of equivalent yarn composition and warp density. The average peak‐to‐peak OCV reached approximately 3 mV for the solo actuating yarn and 4 mV for the auxetic‐integrated configuration (Figure 4E), whereas the satin‐integrated reference exhibited only 0.7 mV (Figure S4). Since the yarn composition, actuator integration procedure, and warp density were kept constant, the observed difference is primarily attributed to the textile architecture. Although modest, the negative Poisson's ratio may have contributed to the enhanced sensitivity by improving strain transfer and mechanical coupling between the electroactive yarn and the folded zig‐zag auxetic structure, potentially reducing relative yarn slippage. The present comparison demonstrates the benefit of the selected auxetic architecture relative to a controlled conventional weave; however, further systematic studies involving different weave geometries and structural parameters will be required to isolate the specific contribution of auxeticity. Notably, the auxetic‐integrated system remained functional after approximately one month under ambient conditions with fluctuating humidity and temperature. Quantitative short‐term stability of the electrochemical system (70 actuation cycles and 1000 sensing cycles) is provided in the Figure S5.

Finally, the full smart textile system was evaluated by simultaneously operating the sensing and actuating modules while worn on the lower biceps. The contractile yarn actuator did not generate a strong, clearly recognizable haptic signal on the skin. This is consistent with the current actuation speed, with a contraction period of approximately 30 s (∼0.03 Hz), which is below the frequency range of rapidly adapting mechanoreceptors (> 0.4 Hz), while the generated forces remain limited relative to tactile perception thresholds [25]. Therefore, the present actuating system is not intended for high‐frequency haptic stimulation, but rather for slow, continuous, and adaptive wearable functions such as thermal regulation, reversible compression, conformal adjustment, or gradual mechanical modulation. In contrast, the sensing module showed excellent performance, reliably tracking real‐time physiological motion. The wireless output accurately captured alternating relaxed–flexed muscle states every 15 s (corresponding to a ∼3% circumference change) (Figure 4F). When both modules were active, high‐frequency switching transients from the actuator introduced minor electrical cross‐talk and ground bounce, shifting the reference potential and amplifying the measured signal (30 mV). This highlights the need for an improved PCB layout without air‐wires, where tightly paired return paths keep current loops small and minimize unintended coupling into neighbouring signal lines.

Nevertheless, the integrated armband demonstrates wireless operation and stable real‐time strain sensing within a textile‐integrated soft bioelectronic platform. Future optimization of the artificial muscle architecture, electrolyte formulation, and electrical driving strategy will be required to increase response speed and extend the application range toward faster feedback modalities.

The biocompatible artificial muscles fabricated from coiled CNT yarn exhibit both actuation and motion‐sensing capabilities. In the present work, two artificial muscles were integrated into the smart textile system to separately emulate these proprioceptive functions. A promising direction for future research would be to achieve simultaneous actuation and sensing within a single artificial muscle by decoupling the millivolt‐scale sensing signal from the volt‐scale actuation input using advanced signal‐processing techniques. Realizing this capability would enable the transition from a dual‐component wearable system to a truly monolithic soft bioelectronic fiber, further enhancing system integration and functionality. To contextualize the performance of the eutectogel‐derived CNT yarn system within the broader artificial muscle landscape, a comprehensive benchmarking matrix is provided in the Table S4.

## 3 Conclusion

This study demonstrates a modular, textile‐compatible platform based on unipolar solid‐state coiled CNT yarn artificial architectures that integrates ionic actuation and ionotronic strain functionalities. This capability is enabled by DES‐based ionic polymer gels incorporating fixed‐charge networks that regulate selective ion transport within the quasi‐solid‐state matrix. Under electrical stimulation, oppositely charged CNT yarns exhibit synchronized contraction, while under mechanical stimulation, the same electrochemical architecture enables the generation of stable open‐circuit voltage (OCV) signals correlated with applied strain. The CNT artificial muscles achieved reversible unipolar actuation with a contractile stroke reaching 2.9% at 90 mN applied force, while in sensing mode it exhibited a strain sensitivity of up to 0.5 mV %^−1^. Poisson–Nernst–Planck modelling confirmed that the sensing response originates from strain‐induced redistribution of mobile ions within the fixed‐charge polymer matrix, extending ion‐mediated electromechanical coupling mechanisms previously observed mainly in liquid‐phase capacitive sensors to a quasi‐solid‐state architecture. The coupled charge variations at the working and counter electrodes result in measurable amplification of the strain‐induced potential difference, enabling stable electrical readout.

To demonstrate textile integration, a bi‐stretch woven substrate with a folded zigzag geometry (hybrid 4/1–1/1–2/2 weave) was engineered to achieve a strain‐dependent negative Poisson's ratio at applied high longitudinal strain (reaching −0.02 at 120%), thereby providing enhanced conformability and mechanical adaptability. Integration of two parallel CNT‐based devices into this auxetic textile resulted in a responsive fabric platform that retained its sensing capability under varying environmental conditions.

Finally, the textile platform was implemented in a battery‐powered wireless armband, controlled by a low‐power ESP32 microcontroller, and evaluated for wearable operation. Although the actuation speed remains below the range required for optimal tactile feedback, highlighting opportunities for future optimization of dynamic performance. The integrated sensing functionality enabled real‐time motion tracking. Overall, this work establishes a scalable connection between unipolar CNT yarn artificial muscles, ionotronic sensing, textile engineering, and ionic polymer materials, providing new opportunities for electroactive textiles, wearable electronics, and adaptive soft robotic systems.

## 4 Materials and Methods

### 4.1 Materials

Carbon nanotube yarns (DexMat, Galvorn CNT yarns, diameter = 150 µm; resistance = 10 Ω/m), polystyrene sulfonate sodium salt (PSS, Mw = 10^6^ g/mol, Aldrich), choline chloride (ChCl, TCI), lithium chloride (LiCl, Janssen Chimica), glycerol (99%, Sigma‐Aldrich), poly(ethylene glycol) diacrylate (PEGDA, Mn = 700 g/mol, Aldrich), 2‐hydroxyethyl acrylate (HEA, Aldrich), 2‐hydroxy‐4′‐(2‐hydroxyethoxy)‐2‐methylpropiophenone (Irgacure 2959, TCI), 2‐acryloxyethyltrimethylammonium chloride (AETA, Aldrich), 3‐sulfopropyl acrylate potassium salt (SPA, Aldrich), textured polyester yarn (Neckelmann, 100167525), polyester polypropylene yarn (Microsilk coslan, Polisilk), polyamide/elastane yarn (n/a), velcro hook (HTH 42A, Velcro), loop fabric (velour, Velcro), nylon tricot jersey (3‐FCAQ Euro Jersey, Takisada Nagoya), Li‐ion battery (3.7 V, EEMB), charging module (TP4056), toggle switch (n/a), microcontroller (XIAO ESP32‐C3, Seeed Studio), resistors (47 Ω, 10 k Ω, Huaban), capacitors (0.1 µF, Huarew).

### 4.2 Fabrication

#### 4.2.1 Treatment and Coiling of the CNT Yarns

The CNT yarns were heat‐treated in air by ramping the temperature to 550°C at a rate of 5°C min^−1^. After thermal activation, the yarns were mounted between a servo motor and a torsionally tethered weight, applying a tensile stress of approximately 20.5 MPa, calculated based on the yarn's cross‐sectional area before coil insertion. During coiling, the yarn length was reduced on average by a factor of 3.8. The process was precisely controlled using an Arduino Uno board to apply the desired turns per meter (T/m) until the onset of overtwisting, corresponding to an average of 3398 T/m. Substituting these values into Equation (5), where β represents the post‐coiling bias angle, D is the yarn diameter, and T is the twist density, yields a theoretical bias angle of 58°, which aligns closely with the experimentally measured average of 65° [36, 37].

(5) β=tan−1(πDT)

#### 4.2.2 Electrolytes

The electrolyte used to supply ions for muscle operation in the gel coatings was an aqueous dilution of a DES composed of glycerol as the hydrogen bond donor (HBD) and choline chloride (ChCl) as the hydrogen bond acceptor (HBA). The weight ratios and molarity of the electrolyte are reported in Table 1.

**TABLE 1:** Composition of the electrolyte.

Name	HBA‐HBD (molar ratio)	HBA‐water (molar ratio)	Molarity (mol/L)	Weight ratio
ChCl 1‐2‐16	1/2	1/16	1.3	23/30/47

#### 4.2.3 Gel Synthesis

The polymer network (PN) precursors were combined with the electrolyte in a 1:1 weight ratio, corresponding to 50 wt.% of the total gel mass. The neutral gel was synthesized via free‐radical copolymerization of 2‐hydroxyethyl acrylate (HEA) as the monomer and polyethylene glycol diacrylate (PEGDA, comprising 4 wt.% of the polymer precursors) as the crosslinker. Polymerization was initiated by 2‐hydroxy‐4′‐(2‐hydroxyethoxy)‐2‐methylpropiophenone (Irgacure 2959, 1 wt.% relative to polymer precursors), a non‐cytotoxic UV‐sensitive initiator [28]. The polyanionic and polycationic gels were prepared following the same procedure, with the HEA monomer replaced, respectively, by 3‐sulfopropyl acrylate potassium salt (SPA), introducing grafted anionic groups (─SO_3_^2−^), and 2‐acryloxyethyltrimethylammonium chloride (AETA), introducing grafted cationic groups (─(N(CH_3_)_3_)^+^). The detailed formulations of all gels used in fabricating the unipolar solid‐state actuator are summarized in Table 2.

**TABLE 2:** Formulation of the gels for the solid‐state actuator.

	Monomers (wt.% from PN)^a^			Crosslinker (wt.% from PN)	Photoinitiator (wt.% to PN)	Electrolyte (wt.% to PN)
	HEA	SPA	AETA	PEGDA	Irgacure 2959	ChCl DES dilute
Neutral gel	96			4	1	100
Polyanionic gel (SPA@90)	—	96	—	4	1	100
Polycationic gel (AETA@90)	—	—	1.25^b^	4	1	100

^a^ Polymer network.

^b^ AETA is in the form of an 80% in water solution.

#### 4.2.4 Fabrication of Coiled Yarn Artificial Muscles

The coiled CNT yarn actuators were fabricated from heat‐treated CNT yarns that were pre‐soaked for 15–30 min in either the polyanionic or polycationic gel precursor solutions. The yarns were then coiled by fixing one end to a servo motor and the other to a torsionally tethered load, applying twist until the onset of overcoiling. The applied tensile stress was approximately 20.5 MPa, calculated based on the yarn diameter prior to coiling. After coiling, the yarns were subsequently coated twice with the same precursor solutions. After each coating step, UV polymerization was carried out directly on the coiling setup for 30–90 s (depending on lamp intensity) to ensure complete crosslinking. The final solid‐state actuators consisted of two oppositely charged coiled yarns coupled in parallel and embedded within a neutral gel matrix. The neutral gel precursor was drop‐cast between the yarns, followed by an additional 90 s UV curing step. For comparison, a control actuator was prepared using the same process but with only the neutral gel for both coating and binding.

#### 4.2.5 Fabrication of Textile‐Based Artificial Muscles

The fabric samples were woven on a 24‐shaft computer‐assisted dobby handloom (ARM 60, Handloom Holdings Ltd, UK), where the warp lifting pattern was digitally controlled via imported peg plans, while the weft was inserted manually. The warp was prepared using a straight draft configuration consisting of 64 yarns, with a 3:1 ratio of elastic to inelastic yarns, arranged at a density of 10 threads per centimeter.

The auxetic‐inspired weave architecture combined a 4/1 twill float pattern, forming the flexible folded regions, with a hybrid plain‐backed twill characterized by 1/1 interlacements in the warp and 2/2 floats in the weft, serving as the more rigid component. The weft was hand‐inserted following a 1:1 elastic‐to‐inelastic yarn sequence. This structural configuration enabled differential extensibility between adjacent fabric segments, facilitating controlled folding and unfolding under applied tensile deformation.

For comparison, a control fabric was woven using the same warp and weft configuration but adopting a satin weave pattern. All yarns were synthetic and chemically inert, ensuring compatibility with the electroactive components and polymer gels used in subsequent actuator integration. The key physical and mechanical properties of the yarns are summarized in Table 3. Two coiled CNT yarn artificial muscles, 15 cm in length, were integrated along the warp direction during weaving, following an over‐4‐wefts and under‐4‐wefts insertion pattern.

**TABLE 3:** Yarns used for the textile substrate.

Yarn	Elastic	Non‐elastic weft	Non‐elastic warp
Composition	Elastane/polyamide (EL/PA)	Polyester (PET)	Polyester/polypropylene (PET/PP)
Young's modulus (MPa)	3.3	294	349
Linear density (dtex)	309	428	203

#### 4.2.6 Fabrication of the Smart Armband

The armband was constructed using three layers. The innermost layer, in contact with the skin, consisted of a nylon tricot jersey knit with a loop fabric attached to its back via scirocco binding. The outermost layer was also made of jersey knit, enclosing the textile structure. The armband measured 30 cm in length and 8 cm in width, with an adjustable 5 cm Velcro closure. All textile components were laser‐cut to ensure dimensional stability and uniformity. Electronic components were attached to the inner loop fabric via Velcro, enabling modular placement, easy replacement, and maintaining washability.

The textile‐based artificial muscle was secured in a pre‐stretched state within a ripstop‐fabric tunnel using hook attachments at both ends. This tunnel allowed simple replacement of the actuator and provided mechanical protection.

The electronic system integrated into the armband consisted of a 3.7 V Li‐ion battery with a TP4056 charging module, a toggle switch for power control, and a Seeed Studio XIAO ESP32‐C3 microcontroller. Two coiled CNT yarn artificial muscles were incorporated: one functioning as an actuator and the other as a strain sensor. For the actuator, the yarns serving as the working electrode (WE) and counter electrode (CE) were connected to two digital output pins (GPIO6 and GPIO7). The sensing yarn was connected to an analog input pin (GPIO3) and ground. Resistors and capacitors were incorporated according to the schematic shown in Figure 4D to ensure signal stability and minimize electrical noise.

### 4.3 Methods

#### 4.3.1 Yarns and Fabric Characterization

The yarn's geometry was characterized by SEM, performed using a Carl Zeiss AG‐ULTRA 55 by GEMINI with a field emission gun at 3 kV, at ∼5 mm distance.

Tensile testing of the yarns was performed on an LS1 Amitek LLOYD tensile tester, and the Young's modulus was determined from the initial slope of the stress–strain curve. Fabric samples were subjected to cyclic stretching at a rate of 10 mm/min up to 120% strain, which is near their elongation at break. The deformation of the fabrics was recorded using a camera, and the resulting videos were analyzed with semi‐automatic motion tracking software (Tracker, Open Source Physics) by tracking a pre‐defined reference point to quantify strain and displacement.

#### 4.3.2 Electrochemical and Electromechanical Measurements

Two coiled CNT yarns forming an artificial muscle were actuated in open air by connecting them respectively to the WE and CE of a potentiostat (Gamry Instruments, Ref 600), and applying a preload of 90 mN. Chronoamperometry (CA) was conducted at potential steps of −3 and 3 V, with 30 s duration per step. Contraction of samples shorter than 2 cm was measured using a force‐displacement lever arm transducer (Aurora Scientific model 300C, Canada), while the strain of longer samples was characterized using video recordings and tracking a reference point via Tracker software (Open Source Physics).

The sensing behaviour of the yarn muscles was characterized through OCV, as each of the two parallel yarns was connected to the WE and the CE, and tensile strain was applied on both ends at different rates, using the LS1 Amitek LLOYD tensile tester.

#### 4.3.3 Artificial Muscle Control in the Stand‐Alone System

The portable actuation–sensing system was powered by a 3.7 V lithium‐ion battery connected through a TP4056 charging protection module and a toggle switch, which controlled the power supply to the microcontroller unit (MCU). A Seeed Studio XIAO ESP32‐C3 served as the core control and acquisition unit, providing both digital and analog interfacing capabilities. Two coiled CNT yarns were integrated into the circuit—one operating as an electrochemical actuator and the other as a strain‐sensing element.

The actuator yarns were connected to two digital output pins on the MCU (GPIO6 and GPIO7). The code defined a polarity variable to toggle the voltage across these pins every 30 000 ms (30 s). When polarity was true, GPIO6 was driven HIGH and GPIO7 LOW, and vice versa when false, effectively reversing the potential across the actuator. This digital switching simulated a square wave voltage supplied by a potentiostat. A 47 Ω resistor was placed in series with the actuator to limit the potential difference to 3 V, ensuring stable and safe operation of the electrochemical actuator.

The sensing yarn's working electrode (WE) was connected to an analog input pin (GPIO3), while the counter electrode (CE) was tied to ground, enabling passive voltage measurement. To stabilize the sensing output, a 10 kΩ resistor and 0.1 µF capacitor were connected in parallel between GPIO3 and GND, forming an RC low‐pass filter that suppressed noise and potential fluctuations. An additional 0.1 µF capacitor was placed between the 3.3 V supply and ground to decouple power‐line interference between the actuator and sensing channels.

Analog sensing was performed using the ESP32‐C3's 12‐bit ADC, configured via analogReadResolution(12) to yield 4096 discrete levels (0–4095). The attenuation was set to 0 dB (analogSetAttenuation(ADC_0db)), which sets the ADC's internal reference voltage to 1.1 V, defining the measurable range as 0–1.1 V, which corresponds to the ADC's most linear region. Each measurement was digitally filtered by averaging 16 consecutive ADC samples, implemented in the readSensorFiltered() function, effectively reducing random fluctuations. The physical voltage (𝑉) was derived from the ADC output using Equation (6):

(6) $$V=\frac{rawReading}{4095}\;*1.1$$

The ESP32‐C3 established a Wi‐Fi connection to a local network and hosted a lightweight HTTP server on port 80. The server endpoint (/) returned the most recent filtered sensor voltage as plain text. A Python‐based data acquisition script periodically queried the MCU every 500 ms, extracted the numerical voltage readings, and plotted them in real‐time using Matplotlib. All time–voltage data pairs were simultaneously logged to a text file for subsequent analysis. This configuration enabled wireless, high‐resolution monitoring of open‐circuit potential variations in the coiled CNT yarn sensor during actuator operation, providing insight into the coupled electrochemical–mechanical response.

## Conflicts of Interest

The authors declare no conflicts of interest.

## Supporting information




**Supporting File 1**: smll75219‐sup‐0001‐SuppMat.docx.

**Supporting File 2**: smll75219‐sup‐0002‐VideoS1.mp4.

## Data Availability

The data that support the findings of this study are available on request from the corresponding author. The data are not publicly available due to privacy or ethical restrictions.
