# Supplementary material for: Dual‐Functionality, Bio‐Friendly Artificial Muscles for Actuation and Motion Sensing in Smart Wearable Systems
Source: Small. 2026 Aug 14;22(59):e75219. doi: 10.1002/smll.75219 (PMC13690615; doi:10.1002/smll.75219)
Supplement: Supplementary file 1 [file SMLL-22-e75219-s001.docx]

Supporting Information

Dual-functionality, Bio-friendly Artificial Muscles for Actuation and Motion Sensing in Smart Wearable Systems

Gabriela Ananieva^1^, Cédric Vancaeyzeele^1^, Giao T.M. Nguyen^1^, Frédéric Vidal^1^, Thuan Nguyen Pham Truong^1^, Nils-Krister Persson^2^, Li Guo^2^, Tommie Varekamp^3^, Felix Dransfeld^3^, Jasper Sikken^3^, Bram Bicknese^3^, Cédric Plesse^1^*

In our previous paper, we demonstrated that symmetric, synchronized actuation of both yarns was achieved in solution (three-electrode system) through ion transport and insertion within the two polymer-coated yarn electrodes.^[1]^ A perfectly synchronized actuation of both yarns was observed at 0.05 mV s^-1^. A slight hysteresis appeared only at the highest scan rate investigated (1 V s^-1^) and only for the yarn undergoing chloride insertion. This difference can be interpreted in terms of the transport properties of the choline and chloride ions in the electrolyte. As a reminder, the electrolyte consists of a hydrated deep eutectic solvent (DES) based on choline chloride (HBA) and glycerol (HBD) in a 1:2 molar ratio, with water added at a ChCl:H₂O molar ratio of 1:16. Compared with neat glyceline (choline chloride – glycerol DES), the addition of water substantially reduces the viscosity while largely preserving the hydrogen-bond network characteristic of the DES, thereby significantly enhancing ionic mobility.^[2]^ Pulsed-field gradient (PFG) NMR studies have shown that the choline cation is the slowest diffusing species in this hydrated DES, whereas glycerol exhibits a slightly higher self-diffusion coefficient and water diffuses considerably faster owing to its weaker involvement in the hydrogen-bond network.^[3,4]^ The relatively slow diffusion of the cholinium cation is attributed to its strong interactions with the surrounding glycerol molecules, which effectively form a hydrogen-bonded cage that hinders both its translational and rotational motion. Studies of the differential microscopic mobility of the DES components further indicate that both the cholinium cation and glycerol are strongly associated with chloride ions through an extended hydrogen-bonding network. This microscopic picture was subsequently confirmed by molecular dynamics simulations, which revealed persistent ion–molecule associations throughout the liquid structure.^[4]^ Although the self-diffusion coefficient of chloride cannot be determined directly by conventional PFG-NMR because of the low sensitivity and quadrupolar nature of ^35^Cl, its transport properties has been estimated using electrochemical and molecular dynamics simulations. These studies indicate that the diffusion coefficient of chloride is of the same order of magnitude as that of the choline cation.^[5]^

Consequently, the nearly identical transport properties of the two involved ions explain the highly synchronized actuation observed at low scan rates. At higher scan rates, however, the finite ion transport kinetics become increasingly important. The slightly larger hysteresis associated with chloride insertion likely reflects subtle differences in ion transport and polymer insertion kinetics that become apparent only under rapid electrochemical cycling at 1 V s^-1^.

**Figure S1** Cyclic voltammetry characterization of the gel-coated CNT yarn artificial muscle. CV measurements were performed at scan rates of 50 and 1000 mV s⁻¹ within a −3.8 to +3.8 V and -5 to +5V potential windows, respectively, to evaluate the electrochemical stability of the system.

**Figure S2 A**) Optical microscopy image of a short 14 mm yarn specimen; **B**) Digital photograph of an extended 15 cm yarn specimen, illustrating the result of manual coating distribution over longer spans

**1. Poisson-Nernst-Planck (PNP) modeling**

The Poisson equation for the electrostatic potential $\varphi(x)$ is given as**:**

$\boldsymbol{-}\left( \boldsymbol{\varepsilon}\boldsymbol{\varphi}^{\boldsymbol{'}}\left( \boldsymbol{x} \right) \right)^{\boldsymbol{'}}\boldsymbol{=}\boldsymbol{z}_{\boldsymbol{1}}\boldsymbol{e}\boldsymbol{c}_{\boldsymbol{1}}\left( \boldsymbol{x} \right)\boldsymbol{+}\boldsymbol{z}_{\boldsymbol{2}}\boldsymbol{e}\boldsymbol{c}_{\boldsymbol{2}}\left( \boldsymbol{x} \right)$	(7)

where $c_{1}(x)$ and $c_{2}(x)$ represent the local concentrations of anions and cations, respectively, $z_{i}$ are their valences ($z_{1}$ = -1, $z_{2}$= +1), $e$ is the elementary charge, and $\varepsilon$ is the local dielectric permittivity. The term $\rho_{f}(x)$ is an additional term included to account for the fixed charges on the polymeric backbone. The fixed charge profile $\rho_{f}(x)$ = −X·tanh(2x) describes the bipolar two-electrode geometry of the cell: the polyanionic working electrode (WE, x = −1) carries net negative fixed charge, and the polycationic counter electrode (CE, x = +1) carries net positive fixed charge, with a smooth transition through the electrolyte-filled centre (x = 0). This antisymmetric profile is the minimal continuous representation of the two-electrode cell geometry and avoids artificial sharp discontinuities at the electrode boundaries that would cause numerical instability. The modified Poisson equation becomes:

$-\left( \varepsilon\varphi^{'} \right)^{'}=e(z_{1}c_{1}+z_{2}c_{2}+ \rho_{f}/e)$	**(8)**

The Nernst–Planck equation, which balances ionic diffusion and migration under an electrostatic field, is expressed as:

$\left[ \boldsymbol{-}\boldsymbol{D}_{\boldsymbol{i}}\left( \boldsymbol{c}_{\boldsymbol{i}}\boldsymbol{'}\left( \boldsymbol{x} \right)\boldsymbol{+}\frac{\boldsymbol{z}_{\boldsymbol{i}}\boldsymbol{e}}{\boldsymbol{k}_{\boldsymbol{B}}\boldsymbol{T}}\boldsymbol{c}_{\boldsymbol{i}}\boldsymbol{(x)\varphi'(x)} \right) \right]^{\boldsymbol{'}}\boldsymbol{=0}$,	for $\boldsymbol{i}$= 1, 2	(9)

where $D_{i}$ is the diffusion coefficient of species *i*, $k_{B}$ is the Boltzmann constant, and $T$ is the absolute temperature.^[6]^

At steady-state open-circuit conditions, the total ionic flux vanishes, simplifying to:

$\boldsymbol{c}_{\boldsymbol{i}}\boldsymbol{'}\left( \boldsymbol{x} \right)\boldsymbol{+}\frac{\boldsymbol{z}_{\boldsymbol{i}}\boldsymbol{e}}{\boldsymbol{k}_{\boldsymbol{B}}\boldsymbol{T}}\boldsymbol{c}_{\boldsymbol{i}}\boldsymbol{(x)\varphi'(x)=0}$	(10)

**1.1 Strain coupling and parameter scaling**

Following Chao, Geng, and Krasny,^[6]^ the 1D model was formulated using a set of dimensionless reference parameters:

- bulk ion concentration $c_{o}$
- channel half-length *L*
- characteristic diﬀusion coeﬃcient $D_{o}$
- characteristic potential $\varphi_{0}= k_{B}T/e$
- solvent water permittivity $\varepsilon_{t}$

The resulting equations take the form:

$\boldsymbol{-}\left( \boldsymbol{\varepsilon}\boldsymbol{\varphi}^{\boldsymbol{'}}\left( \boldsymbol{x} \right) \right)^{\boldsymbol{'}}\boldsymbol{=}\boldsymbol{\sigma}_{\boldsymbol{2}}\boldsymbol{(}\boldsymbol{z}_{\boldsymbol{1}}\boldsymbol{c}_{\boldsymbol{1}}\boldsymbol{+}\boldsymbol{z}_{\boldsymbol{2}}\boldsymbol{c}_{\boldsymbol{2}}\boldsymbol{+}\boldsymbol{\rho}_{\boldsymbol{f}}\left( \boldsymbol{x} \right)\boldsymbol{)}$	(11)

$\boldsymbol{c}_{\boldsymbol{i}}\boldsymbol{' +}\boldsymbol{\sigma}_{\boldsymbol{1}}\boldsymbol{z}_{\boldsymbol{i}}\boldsymbol{c}_{\boldsymbol{i}}\boldsymbol{\varphi'=0}$,	for $\boldsymbol{i}$= 1, 2	(12)

where the coefficients are defined as:

$\sigma_{1}=\frac{e\varphi_{0}}{k_{B}T}$,	$\sigma_{2}=\frac{ec_{0}L^{2}}{\varphi_{0}\varepsilon_{t}}$	**(13)**

Here, $\sigma_{1}$ represents the ratio of electrical to thermal energy, and $\sigma_{2}$ scales the charge–potential coupling strength.

Further, the full operator - $(\varepsilon\varphi')'$ was simplified to - $\varepsilon\varphi''$under the assumption that permittivity $\varepsilon$ is spatially uniform ($\varepsilon^{'}\left( x \right)=0$).

However, it was allowed to vary with strain according to $\varepsilon=\varepsilon_{0}\left( 1-0.2*strain \right)$, representing a 2% decrease in dielectric screening at 10% strain, consistent with solvent loss and polymer chain alignment during stretching.

The fixed charge density increases proportionally with strain $X=X_{base}\left( 1+strain \right)$, reflecting expulsion of mobile cations and thereby densification of the charge in the fixed network.

**1.2 Open-Circuit Voltage Calculation**

The coupled system of six first-order ordinary differential equations,

$y=\left[ \varphi,\varphi^{'},c_{1},c_{1}^{'},c_{2},c_{2}^{'} \right],$	**(14)**

was solved numerically using the *scipy.integrate.solve_bvp* solver with adaptive meshing and solution continuation (each strain level used the previous solution as an initial guess).

Boundary conditions were set as:

$\varphi\left( -L \right)=\varphi\left( L \right)=0$**,**	$c_{1}\left( -L \right)=c_{1}\left( L \right)=c_{2}\left( -L \right)= c_{2}\left( L \right)=1$	**(15)**

Once the coupled equations are solved across the domain, the OCV is obtained as the potential difference between the averaged potentials near the working- and counter-electrode sides:

$OCV=(\left\langle\varphi\right\rangle_{WE-side}- {\langle\varphi\rangle}_{CE-side})\varphi_{0}$	**(16)**

where $\varphi_{0}=25.7$ mV is the thermal voltage at 298 K. This avoids using the sharp interfacial values and gives a physically meaningful bulk-to-bulk potential difference. Averaging over bulk regions (|x| ≈ 0.8–1) avoids sharp interfacial gradients and yields a physically representative potential difference. The only parameter tuned to fit the experimental OCV was the base fixed charge magnitude $X_{base}$- a standard computational practice.

**1.3 Parameter mapping and computational implementation**

Table S1 Parameter interpretation from the used equations to python modeling

| Symbol | Meaning | Code variable | Typical value | Comment |
| --- | --- | --- | --- | --- |
| $\boldsymbol{z}_{\boldsymbol{1}}\boldsymbol{,}\boldsymbol{z}_{\boldsymbol{2}}$ | Ion valences | $z_{1}$*= -1,* $z_{2}$*= 1* | --- | Monovalent ions |
| $\boldsymbol{c}_{\boldsymbol{1}}\boldsymbol{,}\boldsymbol{c}_{\boldsymbol{2}}$ | Local ion concentrations | $c_{1}, c_{2}$ | Nondimensional | Initially 1 (bulk) |
| ε | Dielectric permittivity | *eps = eps0*(1-0.2*strain)* | 100 → 98 | Used as a lumped, non-dimensional fitting parameter to account for the combined dielectric screening effects of the polymer network and the water-glycerol solvent within the gel |
| $\boldsymbol{\rho}_{\boldsymbol{f}}\left( \boldsymbol{x} \right)$ | Fixed charge density | *rho_f = -X * np.tanh(2*x)* | −79.6 → −87.6 | Nondimensional parameter, negative for the polyanionic gel |
| $\boldsymbol{\sigma}_{\boldsymbol{1}}$ | $e\varphi_{0}/k_{B}T$ | *sigma1 = 1* | ≈1 | Nondimensional mobility scale |
| $\boldsymbol{\sigma}_{\boldsymbol{2}}$ | $ec_{0}L^{2}/\varphi_{0}\varepsilon_{t}$ | *sigma2 = 5.0* | ---- | Nondimensional value to provide appropriate coupling between charge density and potential |
| $\boldsymbol{\varphi}_{\boldsymbol{0}}$ | Thermal voltage | *phi0 = 25.7e-3* | 25.7 mV | Conversion to physical volts |
| *L* | Domain half-length | *L = 1* | Normalized | Corresponds to 1 cm physically |
| $\boldsymbol{X}_{\boldsymbol{base}}$ | Base fixed charge magnitude | *X_base = 79.6* | Tuned | Nondimensional parameter empirically tuned to match the experimentally observed baseline |
| Strain | Mechanical stretch | *strain_values= 0 - 0.10* | 0 - 10 % | Independent variable |
| OCV | Open-circuit voltage | *Computed* | 7 - 10 mV | Scaled to experiment |

**Physical picture:**

Stretching the polyanionic yarn leads to transverse densification of the electrode, reducing solvent content and accessible pore volume.

This causes partial expulsion of mobile cations from the gel while the immobile fixed anionic sites remain.

As a result, charge screening decreases, the internal electrostatic potential rises, and the measured OCV increases with strain.

In the numerical implementation, these effects are captured implicitly: increasing $\rho_{f}$ and slightly decreasing ε reproduce the reduced cation concentration $c_{2}$ within the polyanionic region and yield an OCV trend consistent with the experimental data.

The raw OCV output from the PNP model (cnt_mV_raw) is nondimensional in nature and depends on parameters like ε, $X_{base}$, and $\sigma_{2}$ that are only approximately known for the experimental system. Because these parameters lump multiple physical effects (e.g., polymer dielectric constant, local ion activity, electrode geometry), the absolute OCV value may differ from the experimental measurement by a constant offset or scale factor. The code performs a linear normalization of the simulated OCV range so that the starting point (0 % strain) corresponds to 7 mV and the end point (10 % strain) corresponds to 10 mV, with the shape and slope of the predicted curve remaining unchanged.

The full Python script can be downloaded as a separate .py file in addition to the other supplementary files.

To verify the key qualitative result - a monotonically increasing OCV with strain for the polyanionic *vs*. the polycationic gel-coated electrodes - the X_base was varied by ±50% and $\sigma_{2}$ , $\varepsilon_{0}$across one order of magnitude (Table S2). The sensitivity analysis confirms that the monotone-increasing OCV-strain relationship is a robust feature of the model that is preserved across the entire parameter space tested. Varying X_base by ±50% and $\sigma_{2}$ and $\varepsilon_{0}$ each across one order of magnitude changes only the absolute magnitude of the OCV, not its direction or qualitative trend. The proportional scaling of OCV with X_base and $\sigma_{2}$, and its inverse scaling with $\varepsilon_{0}$, is fully consistent with the analytical structure of the Poisson equation ($\varphi''$ = −$\sigma_{2}\rho_{f}$/$\varepsilon$), which predicts that the potential curvature - and hence the bulk OCV - scales as X_base*$\sigma_{2}$ / $\varepsilon_{0}$ in the linear regime. This proportionality confirms that the model is operating within a physically well-behaved linear response regime across the strain range studied (0–10%), and that the mechanistic conclusion - strain-induced increase in uncompensated fixed charge density and decrease in dielectric screening together drive a monotonically increasing OCV - is not an artefact of any specific parameter choice.

Table S2 Sensitivity analysis of the PNP model OCV output to key model parameters

|  | OCV at 0% strain | OCV at 10% strain | Trend |
| --- | --- | --- | --- |
| X_base (baseline) | 7 | 7.9 | Monotone increase |
| X_base -50% | 3.5 | 3.9 | Monotone increase |
| X_base + 50% | 10.5 | 11.8 | Monotone increase |
| $\boldsymbol{\sigma}_{\boldsymbol{2}}$*0.5 | 3.5 | 3.9 | Monotone increase |
| $\boldsymbol{\sigma}_{\boldsymbol{2}}$*2 | 14 | 15.7 | Monotone increase |
| $\boldsymbol{\sigma}_{\boldsymbol{2}}$*5 | 35 | 39 | Monotone increase |
| $\boldsymbol{\varepsilon}_{\boldsymbol{0}}$*0.5 | 14 | 15.7 | Monotone increase |
| $\boldsymbol{\varepsilon}_{\boldsymbol{0}}$*2 | 3.5 | 3.9 | Monotone increase |

*All values represent raw PNP output prior to linear rescaling to the experimental 7–10 mV range. The baseline configuration (X_base = 79.6, σ₂ = 5.0, ε₀ = 100) corresponds to the parameters used in the main text.

**Vector B**

**Vector A**

**Figure S3. A)** Normalized lateral strain to time per cycle, corresponding to 120% longitudinal strain under strict boundary conditions, which artificially constrain the absolute magnitude of the transverse expansion due to localized clamping forces (vector A in blue, vector A in black). **B)** Sequential images of the auxetic fabric under more relaxed boundary conditions during longitudinal extension (≈1%, 50%, 90%, and 120% strain). The sample exhibits visible transverse expansion as the zigzag geometry unfolds. The apparent auxetic effect results from the combined influence of elastic recovery of the weft and geometric decrimping, highlighting the role of boundary constraints in the observed behavior.

The physical validity of the relatively small (-0.02 and -0.03) absolute value of the calculated Poisson's ratio (ν)(PR) is rooted in its reproducibility across multiple tensile testing cycles. As shown in **Figure S3A**, the normalized lateral strain per cycle (120% longitudinal stretch) remains strictly positive, thereby confirming a true negative Poisson's ratio (NPR). The absolute magnitude of this lateral expansion is characteristically constrained by the rigid boundary conditions imposed by the yarn clamps during mechanical stretching. This testing configuration was intentionally implemented to isolate the true structural float-flattening mechanism of the weave and strictly eliminate potential geometric artifacts, such as the simple unconstrained decompression of the weft yarns.

To rigorously quantify this behavior, the reported PR was derived from the average elongation of vectors A and B across consecutive stabilized cycles. Statistical analysis of these experimental cycles yielded a mean PR of -0.027±0.004, and a standard error of the mean (SEM) of 0.0019. The corresponding 95% confidence interval (CI) was determined to be [-0.03, -0.02]. Because the entire confidence interval lies strictly below zero, the NPR is demonstrated to be statistically distinct from zero (p < 0.01). This mathematically confirms that the measured auxetic response cannot be attributed to measurement noise or experimental error, but rather constitutes an intrinsic, reproducible mechanical property of the engineered weave architecture.

To further elucidate this behavior under unconstrained states, sequential optical analysis of the fabric was conducted under relaxed boundary conditions during longitudinal extension (~1%, ~50%, ~90%, and ~120% strain), as presented in **Figure S3B**. In this more compliant state, the sample exhibited a highly visible macro-scale transverse expansion as the characteristic zigzag geometry unfolds. This pronounced auxetic effect is driven by the synergistic influence of the elastic recovery of the weft and structural geometric decrimping. To further contextualize the measured Poisson’s ratio, representative values reported for woven auxetic structures are summarized in **Table S3**. The comparison shows that the measured NPR values are within the range reported for macro-scale woven auxetic fabrics, where the magnitude is strongly dependent on weave geometry, yarn composition, and testing direction.

**Table S3** Poisson’s ration of auxetic weaves (Zulifqar, 2018) ^[7]^

| **Stretch-ability** | **Fabric geometry** | **Details** | **Longitudinal stretch direction** | **Max NPR** | **SD** |
| --- | --- | --- | --- | --- | --- |
| Uni-stretch | Fabric with oblique  folded strips |  | n/a | **-0.045** |  |
|  | Fabric with rotating  rectangles geometry |  | n/a | **-0.05** |  |
|  | Fabric with re-entrant  hexagon geometry |  | n/a | **-0.1** |  |
|  | Fabric with parallel inphase  zig-zag folded  strips | 50/50 elastic/non-elastic warp yarns | Transverse to the zig-zag (weft) | **-0.17** |  |
|  |  | 50/50 elastic/non-elastic warp yarns | Parallel to zig-zag (warp) | **-0.1** |  |
| Bi-stretch | Fabric with parallel inphase  zig-zag folded  strips | Plain weave + satin 4/1; 50/50 elastic/non-elastic weft yarns | Transverse to the zig-zag (weft) | **-0.078** | **0.022** |
|  |  |  | Parallel to zig-zag (warp) | **-0.056** | **0.019** |
|  |  | Plain weave + satin 4/1; 100 elastic weft yarns | Transverse to the zig-zag (weft) | **-0.09** | **0.037** |
|  |  |  | Parallel to zig-zag (warp) | **-0.031** | **0.08** |
|  |  | Plain weave + twill 3/1; 50/50 elastic/non-elastic weft yarns | Transverse to the zig-zag (weft) | **-0.095** | **0.025** |
|  |  |  | Parallel to zig-zag (warp) | **-0.066** | **0.019** |
|  |  | Plain weave + twill 3/1; 100 elastic weft yarns | Transverse to the zig-zag (weft) | **-0.12** | **0.036** |
|  |  |  | Parallel to zig-zag (warp) | **-0.032** | **0.018** |
|  |  | Plain weave + twill 2/2; 50/50 elastic/non-elastic weft yarns | Transverse to the zig-zag (weft) | **-0.078** | **0.023** |
|  |  |  | Parallel to zig-zag (warp) | **-0.058** | **0.022** |
|  |  | Plain weave + twill 2/2; 100 elastic weft yarns | Transverse to the zig-zag (weft) | **-0.065** | **0.025** |
|  |  |  | Parallel to zig-zag (warp) | **-0.029** | **0.016** |

**Figure S4. A**) Picture of the satin weave with integrated artificial yarn muscles; **B**) Sensing behavior of the same muscle embedded in a satin weave with warp density and yarns identical to the ones used for the auxetic weave

**Figure S5** **A**) Actuation strain of the unipolar artificial muscle during 70 consecutive cycles in ambient conditions with unregulated humidity and temperature; **B**) and **C**) respectively, current and and charge of the artifical muscle during the same measurement; **D**) Cyclic stability of the artificial muscle, when acting as a sensor (10% strain at 0.5 Hz), presenting 150 cycles out of 1000 (at varying humidity ~40%), and additional 10 cycles after reconditioning the sample at higher humidity (75%) for 4 h.

To evaluate the operational stability of the eutectogel-based coiled CNT yarn system under repeated mechanical and electrochemical loading, cycling characterisation was performed on representative actuator samples under ambient laboratory conditions without active humidity or temperature control.

**Figure S5A–C** presents the actuation stability of a unipolar artificial muscle, subjected to 70 consecutive actuation cycles under ambient conditions. As shown in **Figure S5A**, the contractile stroke remained stable throughout, varying within a narrow range of 0.77–0.82%, with no systematic degradation trend observed across the measurement window. The corresponding current (**Figure S5B**) and stored charge (**Figure S5C**) remained nearly constant across all cycles, confirming stable ionic conduction and consistent double-layer charging behaviour throughout repeated actuation. A slight initial increase in stored charge during the early cycles is consistent with mild electrochemical conditioning behaviour, commonly reported in gel-polymer electrochemical systems as the electrode-electrolyte interface progressively equilibrates upon initial cycling. This effect stabilises rapidly and does not impact the actuation performance in subsequent cycles.

**Figure S5D** presents the sensing stability of the unipolar artificial muscle. The OCV-based sensing response was evaluated over 1,000 continuous tensile cycles under ambient laboratory conditions (~40% RH, ~20 °C). For clarity of presentation, 150 representative cycles are shown in **Figure S5D**, where the initial OCV variation was ~7.6 mV, following a reduction during continuous cycling at these low humidity conditions (frequency of 0.1 Hz). The average OCV variation reaches the values, mentioned in the main text (3 mV) under the same applied strain of 10% at 0.5 Hz. To assess resilience to humidity variation — a practically relevant stress condition for any wearable electrochemical device — the same sample was subsequently stored in a humidity chamber at 75% RH for 4 hours and retested. The OCV variation measured over 10 cycles following this reconditioning step was initially 7.9 mV, representing a difference of less than 4% relative to the pre-conditioning value. This result demonstrates that, while ambient humidity modulates ionic mobility within the gel network and may introduce minor baseline fluctuations, the underlying electromechanical sensing mechanism remains stable, repeatable, and resilient across the humidity range relevant to wearable skin-contact applications.

Systematic long-term stability characterisation over extended cycling (>10,000 cycles) and prolonged storage, including quantitative degradation metrics and failure mode analysis, is identified as a dedicated next step toward device-level deployment and is beyond the scope of the present proof-of-concept study.

**Table S4. Comprehensive comparison of artificial muscle and electroactive actuator technologies.**

| **Parameter** | **Shape memory alloys (SMA (NiTi))** | **Electronic EAP — Dielectric elastomers (DEA)(fiber shaped)** | **Ionic EAP — Conducting Polymer (PPy/PEDOT)** | **CNT yarn — organogel electrolyte (open air)** | **CNT yarn — hydrogel electrolyte (open air)** | **This work — Eutectogel-derivative CNT yarn (open air)** |
| --- | --- | --- | --- | --- | --- | --- |
| **Actuation mechanism** | Thermally induced martensitic phase transformation | Maxwell electrostatic pressure compresses dielectric, causing radial contraction and axial extension | Redox-driven ion insertion/expulsion causing volumetric change | EDL capacitive ion intercalation in gel-encapsulated coiled yarn | EDL capacitive ion intercalation in gel-encapsulated coiled yarn | EDL capacitive ion intercalation in DES-based eutectogel-coated unipolar coiled yarn |
| **Contractile strain** | 4–8% (wire); up to 55% (spring/fabric) | <10% | <1% (linear) | Up to 18.3% | 1-3.9% | Up to 3.4% |
| **Working voltage** | N/A (thermal); Joule heating via current | kV range (2.5–8 kV for fiber) | 1–5 V | 1–5 V | 1-3 V | 3 V |
| **Actuation speed** | Slow (limited by thermal cycle and cooling); <1 Hz passive cooling | Fast (ms response); up to 10 Hz | <0.1 %/s typical | Up to 3.6 %/s | Up to 0.78 %/s | Up to 0.21 %/s |
| **Max stress output** | High (100–700 MPa); among highest of all actuators | 4 – 20 MPa | Low–moderate (<5 MPa) | Up to 37 MPa | Up to 12.5 MPa | 5.5 MPa |
| **Specific volumetric work capacity*** | <40 000 kJ/m^3^ | <1000 kJ/m^3^ | ~40 kJ/m^3^ | ~ 5000 kJ/m^3^ | 500 kJ/m^3^ | 120 kJ/m^3^ |
| **Sensing capability** | No intrinsic sensing; requires separate strain gauge | ✅Capacitive self-sensing possible (same electrode) | Not demonstrated in open air | Not demonstrated in open air | Not demonstrated in open air | ✅Yes — OCV strain sensing in open air (this work) |
| **Sensing signal** | - | n/a |  |  |  | OCV 7–10 mV per 10% strain, 0.5 Hz (open air, ambient) |
| **Open-air stability** | ✅ (thermal, no electrolyte needed) | ✅ (solid dielectric, no electrolyte) | Partial (gel electrolyte required; drying is an issue) | Gel prone to drying without additional encapsulation | Gel prone to drying without additional encapsulation | ✅ Performance back to baseline after re-hydration in higher humidity conditions |
| **Biocompatibility** | Moderate (NiTi: Ni ion release concerns) | ✅Yes (silicone matrix) | ✅High (PPy, PEDOT biocompatible) | No | ✅Yes | ✅Yes (DES components: glycerol, choline chloride — GRAS) |
| **Textile integration** | Partial (weavable wire; stiff; Joule heating required) | Possible, but kV wiring incompatible with garments | Partial (film or fiber format; fragile) | ✅ Yarn format; weavable | ✅ Yarn format; woven demonstrated | ✅ Woven into auxetic textile architecture demonstrated |
| **Fabrication complexity** | Low (commercial wire available) | Moderate (multi-layer co-extrusion,  <10 m scale) | Moderate (electropolymerisation required) | High (complex yarn architectures + gel synthesis + coating) | High (complex yarn architectures + gel synthesis + coating) | ✅Low - moderate (industrial yarns + gel synthesis + coating) |
| **Key limitation** | Slow cooling; high temperature required; Carnot-limited efficiency | kV driving voltage incompatible with wearables; dielectric breakdown risk | Low strain; poor cycling stability; slow | Toxicity, volatility, flammability, lack of air stability | High fabrication complexity, lack of air stability | Low actuation speed |
| **Key advantage** | Highest stress output; commercially mature | Fast response; air-stable; no electrolyte bath | Biocompatible; low voltage; flexible | High speed, stroke and stress output | High speed and stress output, relatively high stroke | Higher stability than typical hydrogels, dual-functionality |
| **Reference** | ^[8,9,10]^ | ^[11,12]^ | ^[47][48]^ ^[13,14]^ | ^[15,16,17]^ | ^[18]^ |  |

** Volumetric work capacity estimates derived from peak stress and strain values.*

*_________________________________*

***References***

[1] G. Ananieva, C. Vancaeyzeele, G. T. M. Nguyen, D. Aguilera‐Bulla, M. Pinault, F. Vidal, C. Plesse, *Adv. Funct. Mater.* **2026**, 36, 1

[2] A. Yadav, S. Trivedi, R. Rai, S. Pandey, *Fluid Phase Equilib.* **2014**, 367, 135

[3] I. Delso, C. Lafuente, J. Muñoz-Embid, M. Artal, *J. Mol. Liq.* **2019**, 290, 111236

[4] D. V Wagle, G. A. Baker, E. Mamontov, *J. Phys. Chem. Lett.* **2015**, 6, 2924

[5] S. Spittle, D. Poe, B. Doherty, C. Kolodziej, L. Heroux, M. A. Haque, H. Squire, T. Cosby, Y. Zhang, C. Fraenza, S. Bhattacharyya, M. Tyagi, J. Peng, R. A. Elgammal, T. Zawodzinski, M. Tuckerman, S. Greenbaum, B. Gurkan, C. Burda, M. Dadmun, E. J. Maginn, J. Sangoro, *Nat. Commun.* **2022**, 13, 219

[6] Z. Chao, W. Geng, R. Krasny, *J. Comput. Electron.* **2023**, 22, 1396

[7] A. Zulifqar, *Study of Auxetic Woven Fabrics, Hong Kong Polytechnic University*, **2019**

[8] M. Kim, J. Heo, H. Rodrigue, H. Lee, S. Pané, M. Han, S. Ahn, *Adv. Mater.* **2023**, 35, DOI 10.1002/adma.202208517

[9] R. Granberry, K. Eschen, B. Holschuh, J. Abel, *Adv. Mater. Technol.* **2019**, 4, DOI 10.1002/admt.201900548

[10] J. Abel, J. Luntz, D. Brei, *Smart Mater. Struct.* **2012**, 21, 085011

[11] Z. Kang, L. Yu, Y. Nie, M. Skowyra, S. Zhang, A. L. Skov, *Adv. Funct. Mater.* **2024**, 34, DOI 10.1002/adfm.202314056

[12] I. A. Anderson, T. A. Gisby, T. G. McKay, B. M. O’Brien, E. P. Calius, *J. Appl. Phys.* **2012**, 112, DOI 10.1063/1.4740023

[13] Y. Kim, Y. Yoshida, *Polymers.* **2023**, 15, 3455

[14] J. Arias-Pardilla, T. F. Otero, J. G. Martínez, Y. A. Ismail, in *Asp. Fundam. Appl. Conduct. Polym., InTech,* **2012**

[15] Y. Wang, J. Qiao, K. Wu, W. Yang, M. Ren, L. Dong, Y. Zhou, Y. Wu, X. Wang, Z. Yong, J. Di, Q. Li, *Mater. Horizons* **2020**, 7, 3043

[16] J. Wang, S. Zhang, Z. Ma, L. Yan, *Green Chem. Eng****.* 2021**, 2, 359

[17] J. A. Lee, N. Li, C. S. Haines, K. J. Kim, X. Lepró, R. Ovalle‐Robles, S. J. Kim, R. H. Baughman, *Adv. Mater.* **2017**, 29, 1700870

[18] H. Chu, X. Hu, Z. Wang, J. Mu, N. Li, X. Zhou, S. Fang, C. S. Haines, J. W. Park, S. Qin, N. Yuan, J. Xu, S. Tawfick, H. Kim, P. Conlin, M. Cho, K. Cho, J. Oh, S. Nielsen, K. A. Alberto, J. M. Razal, J. Foroughi, G. M. Spinks, S. J. Kim, J. Ding, J. Leng, R. H. Baughman, *Science.* **2021**, 371, 494
